# Duration-dependent hippocampal structural changes in focal epilepsy: multicenter neuroimaging evidence

**DOI:** 10.1186/s12967-026-08230-x

**Published:** 2026-05-08

**Authors:** Yuxin Wu, Zaiyu Zhang, Baohui Yang, Junyi Tan, Yue Qin, Chenyang Li, Yujie Zhu, Xinping Luan, Hongmei Han, Yasi Yang, Zi Yang, Shihang Chen, Hui Gan, Jing Zhao, Xuan Zhai

**Affiliations:** 1 Department of Neurosurgery, Children’s Hospital of Chongqing Medical University, National Clinical Research Center for Child Health and Disorders, Ministry of Education Key Laboratory of Child Development and Disorders, No. 136, Zhongshan 2nd Road, Yuzhong District, Chongqing, 400010 China; 2 Chongqing Key Laboratory of Translational Medical Research in Cognitive Development and Learning and Memory Disorders, Chongqing, China; 3 Center for Neuroscience Research, School of Basic Medicine, Chongqing Medical University, Medical College Road 1, Chongqing, Yuzhong District 40016 China; 4 Second Affiliated Hospital of Xinjiang Medical University, Urumqi, Xinjiang 830063 China; 5 Department of Neurology, Epilepsy Center, Lanzhou University Second Hospital, Lanzhou University, Lanzhou, China

**Keywords:** Epilepsy, Hippocampal sclerosis, Disease duration, Neuroimaging, Morphometry, Longitudinal study

## Abstract

**Background:**

Hippocampal sclerosis (HS) represents a higher proportion of surgical pathology in adult epilepsy patients compared to pediatric surgical cohorts. We hypothesized that while HS may serve as an initial pathological substrate, hippocampal abnormalities in epilepsy may also represent a duration-related structural change process that evolves with disease progression. To test this duration-dependent hypothesis, we investigated associations between disease duration and hippocampal morphological features using advanced neuroimaging in a large multicenter cohort.

**Methods:**

This study included 705 patients with unilateral focal epilepsy (age 25.4 ± 14.9 years, disease duration 14.8 ± 13.6 years) and 424 healthy controls from three centers in China. Global hippocampal features (volume, thickness, gyrification, mean curvature, intrinsic curvature) and subfield volumes were extracted using HippUnfold and AID-HS. Z-scores were calculated using normative models controlling for age, sex, total intracranial volume, and site. Associations between disease duration and hippocampal features were assessed using Spearman correlations and duration-stratified group comparisons. Longitudinal changes were evaluated in 80 patients with serial MRI scans using linear mixed-effects models.

**Results:**

Patients with epilepsy showed significant bilateral hippocampal abnormalities compared to controls, with more severe changes ipsilateral to the seizure focus. Disease duration significantly correlated with all ipsilateral hippocampal metrics: negatively with volume (ρ = −0.181, *p* < 0.001), thickness (ρ = −0.135, *p* < 0.001), and gyrification (ρ = −0.141, *p* < 0.001), and positively with mean curvature (ρ = 0.121, *p* = 0.003) and intrinsic curvature (ρ = 0.171, *p* < 0.001). Contralateral associations were observed for volume (ρ = −0.085, *p* = 0.044), thickness (ρ = −0.082, *p* = 0.049), and intrinsic curvature (ρ = 0.080, *p* = 0.049). Longitudinal analysis revealed significant annual decline in ipsilateral hippocampal volume (β = −0.21 Z-score units/year, *p* = 0.042).

**Conclusions:**

We provide novel neuroimaging evidence supporting the duration-related structural alteration hypothesis of hippocampal structural changes in focal epilepsy. These findings may help explain the higher proportion of HS observed in adult surgical cohorts compared to pediatric surgical cohorts and enhance understanding of epilepsy pathophysiology, suggesting that disease duration is significantly associated with hippocampal structural abnormalities, indicating that it may serve as an important reference indicator for clinical monitoring and therapeutic decision-making.

**Graphical Abstract:**

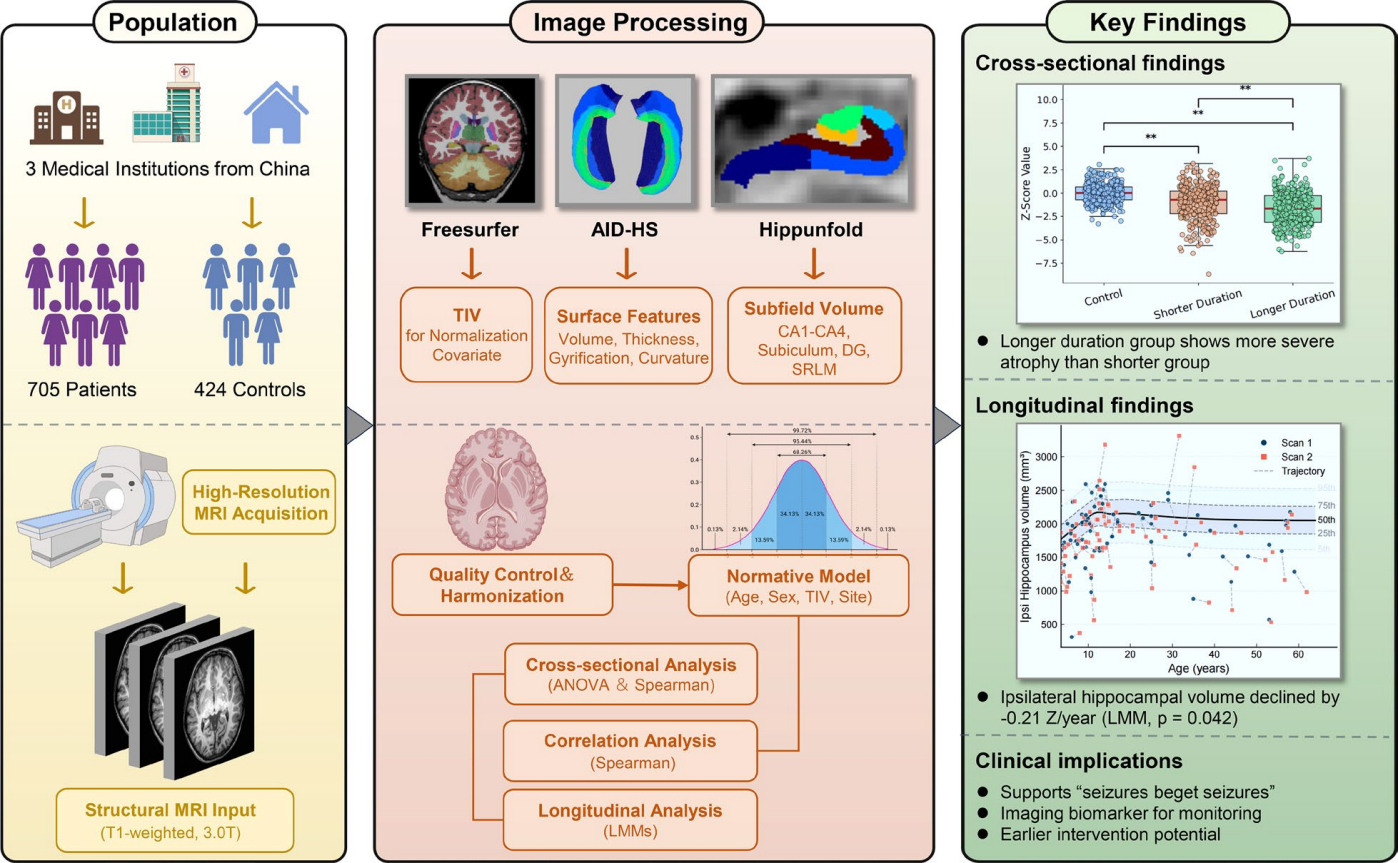

**Supplementary information:**

The online version contains supplementary material available at 10.1186/s12967-026-08230-x.

## Introduction

Epilepsy is a common neurological disorder affecting over 70 million people worldwide [1]. In adult patients with drug-resistant epilepsy, hippocampal sclerosis (HS) represents the most frequent histopathological diagnosis following surgical resection, accounting for approximately 35% of all resected specimens [2, 3]. Histopathologically, HS is characterized by significant neuronal loss and gliosis, predominantly occurring in specific hippocampal subregions (CA1, CA3, and dentate gyrus hilus) [2, 4].

Notably, despite being the most common histopathological diagnosis in epilepsy, HS shows significantly higher detection rates in adult surgical cohorts than in pediatric cohorts. Histopathological analysis of 9,523 patients undergoing epilepsy surgery by the European Epilepsy Brain Bank (EEBB) revealed that HS constituted 44.5% of all pathological types in adult surgical cases, compared to only 15.0% in children [3]. This age-related disparity in HS prevalence admits several explanations: one possibility is that HS represents a primarily adult-onset, age-dependent degenerative process. However, clinical data challenge this hypothesis: although most HS patients undergo surgery in adulthood, 78.1% experienced seizure onset before 18 years of age [3]. An alternative explanation might involve limitations of magnetic resonance imaging (MRI) in detecting early HS, leading to delayed diagnosis and the observed distributional differences [5]. Nevertheless, this explanation remains insufficient, as other lesions that are similarly difficult to detect on MRI (such as focal cortical dysplasia Type I) do not exhibit such pronounced age-related disparities [3, 6, 7].

In 1885, William Gowers’ seminal observation that “seizures beget seizures” proposed an alternative possibility: in at least a subset of cases, HS may not represent the initial pathological substrate of epilepsy, but could emerge over time as a secondary consequence of recurrent seizures, with progressive hippocampal injury and remodeling contributing to sclerosis [8]. From this perspective, HS might serve as both a cause and a consequence of chronic epilepsy. Supporting evidence comes from the “dual pathology” phenomenon: 5–15% of HS patients harbor concurrent brain lesions (such as malformations of cortical development, tumors, or vascular malformations), suggesting that HS can develop secondary to other epileptogenic processes [9–11].

Recent advances in MRI technology have enabled more detailed investigation of structural brain changes in epilepsy. With these technical advances, MRI studies have begun to characterize imaging features associated with HS, including hippocampal atrophy and increased T2/FLAIR signal intensity [12–14]. Building on this progress, several cross-sectional MRI studies have further suggested that longer disease duration may be associated with reduced hippocampal volume [15, 16]. However, despite these advances, the available evidence remains incomplete and sometimes inconsistent, largely because many prior studies were limited by small sample sizes and a lack of longitudinal data [13, 14, 17]. Importantly, previous neuroimaging studies have rarely included the full age spectrum from childhood to adulthood, leaving the developmental trajectory of HS insufficiently understood. In addition, most prior studies treated the hippocampus as a single anatomical structure, thereby overlooking the marked heterogeneity of its subregions in cellular architecture, connectivity, and vulnerability to injury.

Based on these observations, we hypothesized that hippocampal structure in epilepsy patients would progressively evolve toward the characteristic pathological features of hippocampal sclerosis as the disease progresses. To test this hypothesis, we integrated neuroimaging data from epilepsy patients spanning childhood to adulthood across three research centers, utilizing deep learning-assisted high-resolution subfield segmentation to extract quantitative morphological metrics from hippocampal subfields [18, 19]. Through this large-scale, cross-age study design, we aimed to explore the dynamic associations between disease duration and bilateral hippocampal morphological characteristics, providing neuroimaging insights into the pathophysiology of HS development. We also performed exploratory analyses to examine potential medication effects (valproic acid) on hippocampal structure.

## Methods

### Study population

This study consecutively recruited 848 patients with unilateral focal epilepsy from three medical centers in western China. To encompass patients across different age groups, we employed an age-stratified multicenter design: the Chongqing site (pediatric epilepsy specialty center) recruited patients under 18 years of age, while the Lanzhou and Xinjiang sites (comprehensive epilepsy centers) recruited adult patients, enabling observation of hippocampal structural changes across different developmental stages. Brain MRI data were acquired on 3.0T scanners using standardized high-resolution 3D T1-weighted sequences across all sites. Detailed scanner specifications, acquisition parameters, and quality-control procedures are provided in Supplementary Methods S1.

All patients’ seizure types and diagnoses were defined and classified according to the International League Against Epilepsy (ILAE) 2025 updated classification of epileptic seizures [20]. Unilateral focal epilepsy was defined as seizures originating from neural networks limited to one cerebral hemisphere, determined through integration of clinical semiology, electroencephalography (EEG), MRI, and other multimodal data. Each center followed standardized evaluation protocols (including clinical semiology, scalp video-EEG monitoring, high-resolution MRI, and PET/SEEG when necessary) for epileptogenic zone localization, with all cases independently reviewed by experienced multidisciplinary epilepsy teams at each center. Patient exclusion criteria were: (1) unclear seizure type and epileptogenic zone localization; (2) clinical evidence suggesting bilateral epileptogenic zones, generalized epilepsy, or unclassified epilepsy; (3) history of major psychiatric disorders such as schizophrenia or bipolar disorder, or other severe neurological diseases. Cases labeled as ‘undetermined lobe’ refer to patients with confirmed unilateral focal epilepsy in whom precise lobar localization could not be reliably determined, but laterality was established. All participating centers followed the ILAE Neuroimaging Task Force recommendations for the visual diagnosis of hippocampal sclerosis (HS) [21]. To verify the consistency of applying these diagnostic criteria across centers, we performed a center-stratified validation of MRI-based HS diagnosis against histopathological findings in a subset of 277 patients with available hippocampal resection specimens. The analysis demonstrated substantial concordance across all three sites (Cohen’s κ ranged from 0.610 to 0.750; accuracy > 88%). Overall agreement across the validation subset was κ = 0.724 (95%CI 0.626–0.818). Formal inter-radiologist agreement for HS visual diagnosis was not available in this retrospective multicenter dataset, while diagnostic reliability was supported by histopathology validation. Detailed validation methodology and center-specific metrics are provided in Supplementary Methods S1 and Supplementary Table S1.

Given that the core objective of this study was to examine the association between disease duration and hippocampal morphological features, patients with confirmed unilateral focal epilepsy were included even if MRI revealed extrahippocampal structural abnormalities (such as focal cortical dysplasia or encephalomalacia) ipsilateral or contralateral to the epileptogenic zone, provided these abnormalities did not directly involve hippocampal structures or interfere with their imaging assessment. Healthy controls (HCs) were recruited from the same centers and were comparable to patients in age and sex (Table 1). Inclusion criteria for HCs were no history of neurological or psychiatric disorders and no apparent abnormalities on brain MRI examination.

**Table 1:** Demographic and clinical characteristics of participants with focal epilepsy and healthy controls

Characteristic	Focal epilepsy (n = 705)	Healthy controls (n = 424)	p value
Sex, female/male	379/326	235/189	0.629
Age, mean ± SD (years)	25.4 ± 14.9	23.6 ± 17.1	0.086
Onset of epilepsy, mean ± SD (years)	10.5 ± 8.6	NA	NA
Epilepsy duration, mean ± SD (years)	14.8 ± 13.6	NA	NA
Lateralization of seizure focus (n, %)		NA	NA
L	373 (52.9%)	NA	
R	332 (47.1%)	NA	
Localization of seizure focus (n, %)		NA	NA
TL	443 (62.8%)	NA	
FL	163 (23.1%)	NA	
PL	49 (7.0%)	NA	
OL	18 (2.6%)	NA	
IL	3 (0.4%)	NA	
U	29 (4.1%)	NA	
Surgery (n, %)	349 (49.5%)	NA	NA

Demographic and clinical characteristics of participants with focal epilepsy and healthy controls. Data are presented as mean ± standard deviation (SD) or number (percentage) as appropriate. “Onset of epilepsy” refers to the age at first seizure. “Epilepsy duration” refers to the interval between age at seizure onset and age at enrollment.

L, left; R, right; TL, temporal lobe; FL, frontal lobe; PL, parietal lobe; OL, occipital lobe; IL, insular lobe; U, undetermined lobe; NA, not applicable

All participants (or their legal guardians) provided written informed consent prior to study participation. The study protocol was approved by the ethics committees of all participating centers: Children’s Hospital of Chongqing Medical University (Approval No. 2024.yan.111), the Second Affiliated Hospital of Xinjiang Medical University (Approval No. 2022H015), and Lanzhou University Second Hospital (Approval No. D2025-467). The detailed participant screening process is illustrated in Supplementary Figure S1.

### Segmentation and feature extraction

**Fig. 1:**
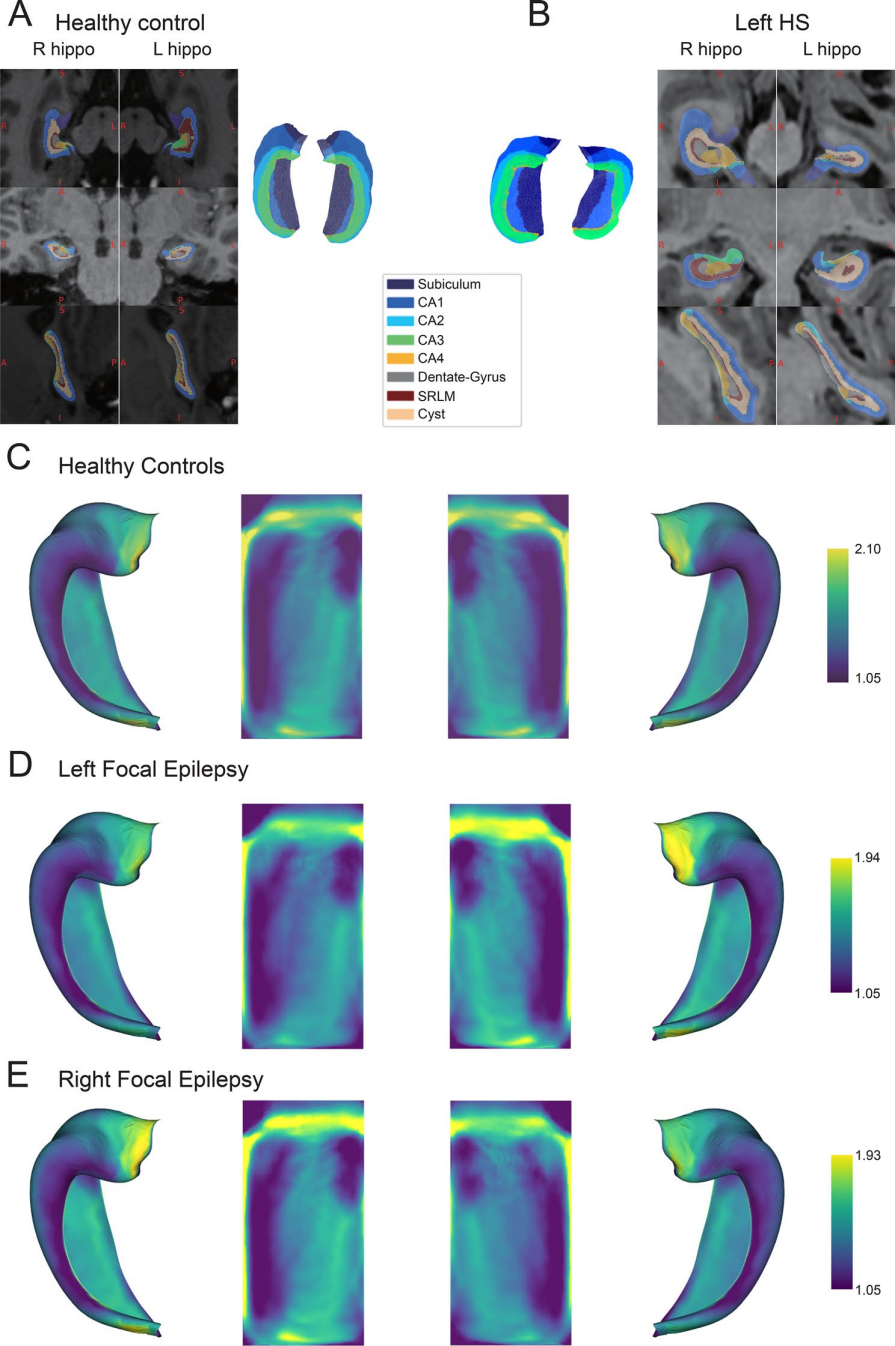
Automated segmentation and surface-based thickness Z-score mapping of hippocampal subfields. (**A**–**B**) automated segmentation of hippocampal subfields in representative individual cases, shown as (**A**) a healthy control and (**B**) a patient with right hippocampal sclerosis (HS). Hippocampal subfields were delineated by HippUnfold (v1.5.2). MR images (left and right hippocampi) are displayed alongside 3D renderings with color-coded subfields: subiculum, CA1, CA2, CA3, CA4, dentate gyrus, stratum radiatum/lacunosum-moleculare (SRLM), and cyst. (**C**–**E**) group-average Z-score maps of hippocampal thickness overlaid on 3D hippocampal surfaces, visualizing the spatial distribution of morphological alterations for each diagnostic group: (**C**) healthy controls, (**D**) patients with left focal epilepsy, and (**E**) patients with right focal epilepsy. Color bars indicate the scale of Z-score deviation from the control mean. HS, hippocampal sclerosis; CA, cornu ammonis subfield; SRLM, stratum radiatum/lacunosum-moleculare; L, left; R, right

To estimate total intracranial volume (TIV), all MRI images were processed using FreeSurfer [22]. We then extracted hippocampal metrics at two scales. For local features, HippUnfold was used for automated segmentation and volume extraction of hippocampal subfields [19]. For global features, the AID-HS toolbox was used to extract surface-based morphological metrics, including total hippocampal volume, cortical thickness, gyrification, and curvature (Fig. 1) [18]. These metrics were selected because hippocampal sclerosis is characterized not only by volume loss, but also by reduced cortical thickness and gyrification together with increased curvature [18, 19].

All segmentation results underwent a rigorous quality control protocol, including independent blinded visual inspection by two neuroradiologists (Cohen’s κ = 0.789) and senior adjudication for non-concordant cases. Detailed descriptions of the software pipelines and quality control procedures are provided in Supplementary Methods S2.

### Z-score standardization and lateralization processing

Prior to normative modeling, hippocampal features were harmonized across sites using the neuroCombat procedure as implemented in AID-HS, to minimize scanner- and site-related variability [18] . To investigate laterality effects, patient data were lateralized by defining the hippocampus ipsilateral and contralateral to the epileptogenic zone. To control potential confounding factors, we first constructed a normative model based on data from the HCs. In this model, hippocampal features were predicted using sex, age, total intracranial volume (TIV), and scanning site as covariates. Because HCs do not have a disease-defined ipsilateral side, both hippocampi were used to construct the age-, sex-, TIV-, and site-adjusted normative reference distribution for Z-score standardization. This bilateral use was intended to improve normative coverage and was used for reference-model construction rather than to define independent subject-level inferential units. We acknowledge that the left and right hippocampi from the same control participant are correlated and therefore not strictly independent observations; accordingly, this strategy may modestly overestimate the effective normative sample size. However, all primary downstream inferential analyses were performed at the patient level using lateralized ipsilateral/contralateral Z-scores or within-subject longitudinal models, rather than by treating bilateral hippocampi from patients as independent observations. This rationale is broadly consistent with prior hippocampal morphometry work that explicitly evaluated hemisphere effects in healthy controls [18, 23].

$${Y_{norm}} = {f_{healthy}}\left( {Age,Sex,TIV,Site} \right)$$

Subsequently, we calculated standardized Z-scores for each hippocampal feature in each patient based on this normative model:

$${Z_i} = \left( {{Y_i} - {{\hat Y}_{i,norm}}} \right)/{\sigma _{norm}}$$

These Z-scores were used for subsequent statistical analyses, reflecting the degree to which individual hippocampal features deviated from the healthy population.

### Data analysis

To evaluate whether duration-related hippocampal abnormalities were consistent across complementary analytical settings, we used a three-stage analytical framework. Stage 1 examined cross-sectional associations between disease duration and hippocampal morphology. Stage 2 evaluated within-individual longitudinal change relative to age-based normative trajectories. Stage 3 assessed the robustness of these associations in supplementary analyses addressing age-related confounding, structural pathology, and medication exposure.

Stage 1: Cross-sectional association analysis. Disease duration was primarily analyzed as a continuous variable using Spearman rank correlations in the full patient cohort. For descriptive visualization of duration-stratified differences, patients were additionally divided into shorter-duration and longer-duration subgroups using the cohort median disease duration (11 years), which provided approximately balanced subgroup sizes (Shorter: *N* = 350; Longer: *N* = 355). This dichotomization was used for descriptive comparison only and not for primary inference [24]. Differences between these subgroups and HCs were assessed using one-way ANOVA followed by post hoc Welch’s t-tests.

Stage 2: Longitudinal validation. To determine whether the cross-sectional associations were consistent with within-individual change over time, we analyzed the longitudinal dataset comprising 80 patients with two MRI examinations. This longitudinal sub-cohort was not derived from a pre-specified prospective protocol; rather, repeat MRI examinations were performed as part of routine clinical care at the participating tertiary epilepsy centers. Common indications for repeat imaging included routine disease surveillance and treatment planning, re-evaluation following changes in seizure control and/or symptom evolution, and imaging updates during pre-surgical evaluation. Among the 705 patients in the cross-sectional cohort, 80 (11.3%) had undergone two MRI examinations that met quality-control criteria and were included in the longitudinal analysis. The mean inter-scan interval was 1.36 ± 1.06 years. To further characterize the temporal sampling of the longitudinal cohort, we summarized the distribution of inter-scan intervals and the distribution of patient-specific annualized slopes for key hippocampal Z-score metrics (Supplementary Figure S5). Individual trajectories were visualized against age-based normative reference curves (Supplementary Methods S3). Longitudinal change in hippocampal Z-scores was then assessed using linear mixed-effects models, with follow-up interval (years) entered as the primary fixed effect. For each feature, the optimal random-effects structure was selected by comparing nested models (random intercept vs. random intercept plus slope) using likelihood ratio tests and Akaike information criterion. The coefficient for follow-up interval in the best-fitting model was interpreted as the annual rate of Z-score change.

Stage 3: Robustness and independence analyses. Although imaging outcomes were standardized using age-based normative models, patient age and disease duration remained inherently correlated. We therefore conducted supplementary analyses to assess the independence of duration effects from age-related confounding, including partial Spearman correlations controlling for age, age-stratified analyses, and formal age × duration interaction tests (Supplementary Methods S4). In addition, we performed prespecified sensitivity analyses to test whether the main associations were robust to structural pathology and medication-related confounding. Specifically, the cross-sectional and/or longitudinal analyses were repeated after excluding (i) patients with MRI-confirmed hippocampal sclerosis and (ii) patients with other structural lesions. To explore whether the duration-dependent associations varied by epilepsy syndrome, we additionally repeated the cross-sectional Spearman correlation analyses after stratifying patients into temporal lobe epilepsy (TLE) and non-TLE subgroups. Additionally, given specific evidence linking valproic acid (VPA) to reversible brain volume loss [25–29], we performed an exploratory medication sensitivity analysis in the subset with complete medication records. Because detailed dose-history data were not consistently available across this retrospective multicenter cohort, patients were classified as valproate users (>6 months), valproate non-users (never exposed), or excluded if exposure was short-term (<6 months), and the duration-dependent models were refitted with valproate exposure included as an additional covariate (Supplementary Methods S5). Other anti-seizure medication classes were not modeled individually because reliable harmonization of class-specific lifetime exposure, cumulative dose, regimen switching, and polytherapy patterns were not feasible in this retrospective multicenter dataset.

In all analyses, *p*-values were corrected for multiple comparisons using the Benjamini–Hochberg false discovery rate procedure, with corrected *p* < 0.05 considered statistically significant. Cross-sectional analyses were performed in R (v4.3.0), and longitudinal mixed-effects models were implemented in Python (statsmodels).

## Results

### Demographic characteristics

This study ultimately included 705 patients with unilateral focal epilepsy and 424 HCs, with both groups well-matched for age and sex composition (see Table 1). The epilepsy patient group had a mean seizure onset age of 10.5 ± 8.6 years and a mean disease duration of 14.8 ± 13.6 years. Within the cohort, 210 patients (29.8%) were diagnosed with hippocampal sclerosis based on visual MRI review, and 52.9% (373 patients) had epileptogenic zones located in the left hemisphere (Table 1). Due to the age-stratified design, patient cohorts recruited from the three centers showed significant differences in age, onset age, and disease duration, with detailed comparisons provided in Supplementary Table S2.

### Hippocampal structural abnormalities in epilepsy patients

Compared to HCs, epilepsy patients demonstrated significant abnormalities across all morphological metrics in the ipsilateral hippocampus. Specifically, total hippocampal volume, cortical thickness, and gyrification index were significantly reduced, while mean curvature and intrinsic curvature were significantly increased. Contralateral hippocampal metrics also showed significant differences from HCs, though the magnitude of changes was typically smaller than ipsilateral alterations (Supplementary Table S3). Sensitivity analysis performed after excluding 210 HS patients revealed that the hippocampal structural abnormality patterns in the non-HS patient subgroup were consistent with the overall cohort, albeit with reduced effect sizes (Table 2), indicating that hippocampal structural changes exist not only in patients with hippocampal sclerosis but also in other unilateral focal epilepsy patients without hippocampal sclerosis. In a medication-documented subset, sensitivity analysis showed that valproate exposure was associated with lower hippocampal Z-scores in several metrics. Importantly, after accounting for valproate exposure, the main duration-related pattern of ipsilateral hippocampal abnormality remained present, suggesting that these associations were not fully explained by valproate exposure alone. However, these results should not be interpreted as establishing independence from all anti-seizure medications. (see Supplementary Methods S5 and Supplementary Table S4-5).

**Table 2:** Group differences in hippocampal Z-Score metrics between focal epilepsy patients without MRI-Diagnosed hippocampal sclerosis and healthy controls

Metric	Mean (SD)	t-statistic	Cohen’s d	p-value (raw)	p value (FDR)
**Total Hippocampus Metrics**					
Ipsi volume	−0.71 (1.66)	−8.00	−0.53	<0.001**	<0.001**
Contra volume	−0.49 (1.48)	−5.96	−0.39	<0.001**	<0.001**
Ipsi thickness	−0.28 (1.35)	−3.60	−0.24	<0.001**	<0.001**
Contra thickness	−0.18 (1.29)	−2.40	−0.16	0.016*	0.018*
Ipsi gyrification	−0.61 (1.66)	−6.79	−0.45	<0.001**	<0.001**
Contra gyrification	−0.34 (1.47)	−4.19	−0.28	<0.001**	<0.001**
Ipsi mean curvature	0.32 (1.62)	3.67	0.24	<0.001**	<0.001**
Contra mean curvature	0.24 (1.31)	3.09	0.20	0.002*	0.002*
Ipsi intrinsic curvature	0.76 (1.94)	7.58	0.50	<0.001**	<0.001**
Contra intrinsic curvature	0.51 (1.81)	5.38	0.36	<0.001**	<0.001**
**Subfield Volumes**					
Ipsi subiculum	−0.63 (1.38)	−7.94	−0.53	<0.001**	<0.001**
Contra subiculum	−0.31 (1.31)	−4.02	−0.27	<0.001**	<0.001**
Ipsi CA1	−0.68 (1.56)	−8.01	−0.53	<0.001**	<0.001**
Contra CA1	−0.49 (1.41)	−6.16	−0.41	<0.001**	<0.001**
Ipsi CA2	−0.60 (1.90)	−6.15	−0.41	<0.001**	<0.001**
Contra CA2	−0.62 (1.64)	−7.03	−0.47	<0.001**	<0.001**
Ipsi CA3	−0.46 (1.68)	−5.07	−0.34	<0.001**	<0.001**
Contra CA3	−0.39 (1.55)	−4.56	−0.30	<0.001**	<0.001**
Ipsi CA4	0.02 (1.51)	0.29	0.02	0.769	0.769
Contra CA4	0.12 (1.39)	1.53	0.10	0.126	0.131
Ipsi DG	−0.49 (1.66)	−5.51	−0.36	<0.001**	<0.001**
Contra DG	−0.27 (1.49)	−3.28	−0.22	0.001*	0.001*
Ipsi SRLM	−0.62 (1.67)	−6.88	−0.46	<0.001**	<0.001**
Contra SRLM	−0.37 (1.48)	−4.48	−0.30	<0.001**	<0.001**

Z-score metrics for hippocampal total and subfield structures comparing focal epilepsy patients without MRI-diagnosed hippocampal sclerosis (HS; *N* = 495) and healthy controls (*N* = 424). All patients with MRI evidence of HS were excluded from this analysis

Group comparisons were performed using Welch’s independent samples t-test. *p* values were corrected for multiple comparisons using the Benjamini-Hochberg False Discovery Rate (FDR) method. Cohen’s d was calculated as d = *t* × √(1/n₁ + 1/n₂)

* *p* < 0.05, ** *p* < 0.001

Ipsi, ipsilateral; Contra, contralateral; CA, cornu ammonis subfield; DG, dentate gyrus; SRLM, stratum radiatum/lacunosum-moleculare

### Laterality effects of hippocampal structural changes

Across the entire patient cohort, paired comparisons revealed that morphological changes in the ipsilateral hippocampus were significantly more severe than those on the contralateral side. Total volume, cortical thickness, gyrification index, and Z-scores for volumes of nearly all subfields were significantly lower ipsilaterally than contralaterally, while curvature Z-scores were significantly higher ipsilaterally (Supplementary Table S6), suggesting more pronounced atrophy and morphological changes more closely resembling hippocampal sclerosis in the ipsilateral hippocampus. In the non-HS patient subgroup, total volume, cortical thickness, gyrification index, and volume Z-scores for subiculum, CA1, DG, and SRLM subfields remained significantly lower ipsilaterally than contralaterally, with intrinsic curvature Z-scores higher ipsilaterally (Table 3), indicating that hippocampal structures ipsilateral to the epileptogenic zone typically exhibit more significant abnormalities than the contralateral side.

**Table 3:** Paired comparison of ipsilateral and contralateral hippocampal Z-score metrics in patients without MRI-diagnosed hippocampal sclerosis

Metric	Ipsi Mean (SD)	Contra Mean (SD)	t-statistic	Cohen’s dₖ	p-value (raw)	p-value (FDR)
**Total Hippocampus Metrics**						
Volume	−0.71 (1.66)	−0.49 (1.48)	−4.36	−0.20	<0.001**	<0.001**
Thickness	−0.28 (1.35)	−0.18 (1.29)	−2.33	−0.10	0.020*	0.030*
Gyrification	−0.61 (1.66)	−0.34 (1.47)	−4.42	−0.20	<0.001**	<0.001**
Mean Curvature	0.32 (1.62)	0.24 (1.31)	1.27	0.06	0.203	0.254
Intrinsic Curvature	0.76 (1.94)	0.51 (1.81)	3.01	0.14	0.003*	0.005*
**Subfield Volumes**						
Subiculum	−0.63 (1.38)	−0.31 (1.31)	−6.27	−0.28	<0.001**	<0.001**
CA1	−0.68 (1.56)	−0.49 (1.41)	−3.09	−0.14	0.002*	0.004*
CA2	−0.60 (1.90)	−0.62 (1.64)	0.17	0.01	0.863	0.863
CA3	−0.46 (1.68)	−0.39 (1.55)	−0.87	−0.04	0.384	0.419
CA4	0.02 (1.51)	0.12 (1.39)	−1.25	−0.06	0.212	0.254
DG	−0.49 (1.66)	−0.27 (1.49)	−3.59	−0.16	<0.001**	<0.001**
SRLM	−0.62 (1.67)	−0.37 (1.48)	−4.29	−0.19	<0.001**	<0.001**

Paired comparison of hippocampal Z-score metrics between ipsilateral and contralateral sides in patients with focal epilepsy who did not have MRI-diagnosed hippocampal sclerosis (*N* = 495). Paired t-tests were used to assess within-subject differences; *p*-values were corrected for multiple comparisons using the Benjamini-Hochberg FDR method. Cohen’s dₖ was calculated as dₖ = t / √N

*p < 0.05, **p < 0.001

Ipsi, ipsilateral; Contra, contralateral; CA, cornu ammonis subfield; DG, dentate gyrus; SRLM, stratum radiatum/lacunosum-moleculare

### Associations between disease duration and hippocampal structural features

Spearman correlation analysis revealed that increased disease duration was significantly associated with decreased ipsilateral hippocampal total volume, cortical thickness, and gyrification index (all P_FDR_ < 0.001), while being significantly associated with increased mean curvature (P_FDR_ = 0.003) and intrinsic curvature (P_FDR_ < 0.001). Further subfield analysis demonstrated that volumes of all subregions, including subiculum, CA1, CA2, CA3, CA4, dentate gyrus, and SRLM, were negatively correlated with disease duration (Table 4). Contralateral hippocampal total volume, cortical thickness, intrinsic curvature, and volumes of some subfields also showed statistically significant associations with disease duration. After excluding patients with MRI-diagnosed hippocampal sclerosis, correlations between disease duration and ipsilateral and contralateral hippocampal total volume, intrinsic curvature, and CA1 volume remained significant (see Supplementary Table S7), indicating that the association between disease duration and hippocampal abnormalities persists after excluding MRI-diagnosed HS and is not solely explained by established HS pathology.

**Table 4:** Spearman correlation between disease duration and hippocampal Z-Score metrics in patients with focal epilepsy

Metric	Spearman ρ	p-value (raw)	p-value (FDR)
**Total Hippocampus Metrics**			
Ipsi volume	−0.181	<0.001**	<0.001**
Contra volume	−0.085	0.024*	0.044*
Ipsi thickness	−0.135	<0.001**	<0.001**
Contra thickness	−0.082	0.030*	0.049*
Ipsi gyrification	−0.141	<0.001**	<0.001**
Contra gyrification	−0.016	0.674	0.702
Ipsi mean curvature	0.121	0.001**	0.003*
Contra mean curvature	0.069	0.066	0.089
Ipsi intrinsic curvature	0.171	<0.001**	<0.001**
Contra intrinsic curvature	0.080	0.033*	0.049*
**Subfield Volumes**			
Ipsi subiculum	−0.151	<0.001**	<0.001**
Contra subiculum	−0.081	0.031*	0.049*
Ipsi CA1	−0.159	<0.001**	<0.001**
Contra CA1	−0.075	0.047*	0.066
Ipsi CA2	−0.155	<0.001**	<0.001**
Contra CA2	−0.014	0.702	0.702
Ipsi CA3	−0.145	<0.001**	<0.001**
Contra CA3	−0.044	0.239	0.287
Ipsi CA4	−0.103	0.006*	0.013*
Contra CA4	−0.032	0.394	0.430
Ipsi DG	−0.134	<0.001**	<0.001**
Contra DG	−0.040	0.289	0.331
Ipsi SRLM	−0.153	<0.001**	<0.001**
Contra SRLM	−0.054	0.149	0.188

Spearman rank correlation coefficients (ρ) between disease duration and hippocampal Z-score metrics in focal epilepsy patients (*N* = 704)

Disease duration was defined as the time from epilepsy onset to enrollment. *p*-values were adjusted for multiple comparisons using the Benjamini-Hochberg False Discovery Rate (FDR) method (α = 0.05)

*p < 0.05, **p < 0.001

Ipsi, ipsilateral; Contra, contralateral; CA, cornu ammonis subfield; DG, dentate gyrus; SRLM, stratum radiatum/lacunosum-moleculare

Because age and disease duration were correlated, we validated the cross-sectional duration analyses using age-adjusted approaches. The Variance Inflation Factor (VIF) for duration was 3.11, indicating no severe multicollinearity. After controlling age, the associations of duration and hippocampal remained directionally consistent, and the negative association with ipsilateral hippocampal volume remained statistically significant (partial Spearman ρ = −0.098, P_FDR = 0.044). The age×duration interaction was not significant after FDR correction (Supplementary Table S8–9; Supplementary Methods S4), suggesting that the impact of disease duration on hippocampal structure is not significantly modified by age. Sensitivity analyses additionally controlling age at seizure onset yielded materially similar results, with partial Spearman correlations remaining nearly unchanged from the unadjusted estimates (Supplementary Table S10), suggesting minimal confounding by onset age.

### Group comparisons based on disease duration

**Fig. 2:**
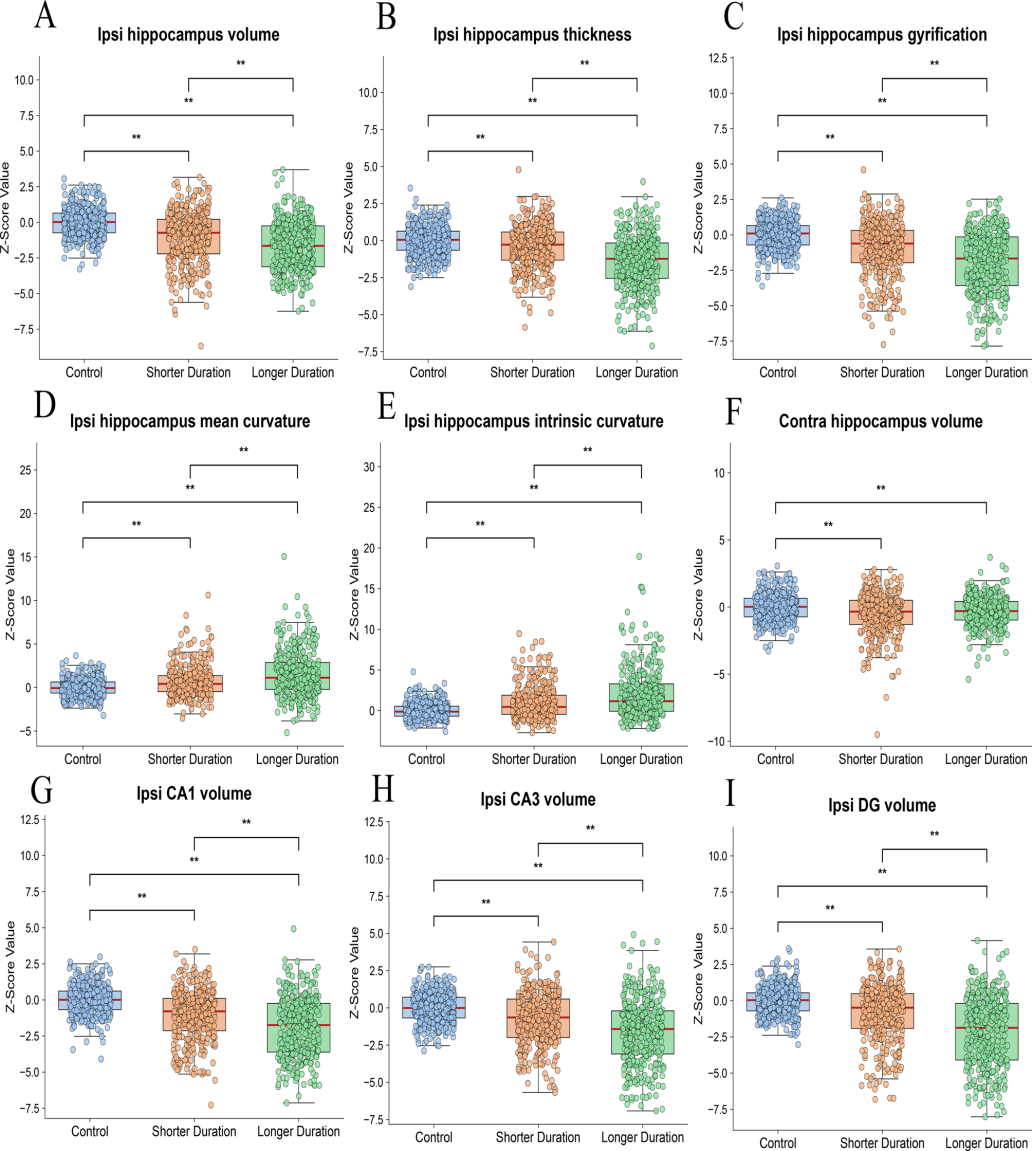
Group comparisons of hippocampal structural Z-scores by disease duration. Boxplots with overlaid individual data points show the distribution of Z-scores for selected hippocampal structural metrics across three groups: healthy controls, patients with shorter disease duration (duration < median), and patients with longer disease duration (duration ≥ median). Panels (**A**–**E**) display global morphological metrics for the ipsilateral hippocampus. Panel (**F**) shows the contralateral hippocampal volume for comparison. Panels (**G**–**I**) display volumes for key ipsilateral hippocampal subfields. Group comparisons were performed using one-way ANOVA with post hoc Welch’s t-tests; *p*-values were corrected for multiple comparisons using the Benjamini-Hochberg false discovery rate (FDR) method. *: *p* < 0.05, **: *p* < 0.001. Ipsi, ipsilateral; Contra, contralateral; CA, cornu ammonis subfield; DG, dentate gyrus; SRLM, stratum radiatum/lacunosum-moleculare

To more intuitively illustrate duration-stratified differences in hippocampal metrics, we divided patients into “shorter duration” (*N* = 350) and “longer duration” (*N* = 355) groups based on the median disease duration and compared them with HCs (Table 5). For the ipsilateral side, all hippocampal morphological metrics showed significant differences among the three groups. Post hoc testing consistently demonstrated that the longer duration group exhibited more severe hippocampal morphological changes compared to the shorter duration group. Specifically, the longer duration group showed significantly lower ipsilateral hippocampal total volume, cortical thickness, and gyrification index compared to the shorter duration group, while mean curvature and intrinsic curvature were significantly higher (all comparisons P_FDR_ < 0.001). This duration-dependent atrophy pattern was observed across global features and representative subfields (CA1, CA3, DG) (Fig. 2), with complete subfield results shown in Supplementary Figure S3. For the contralateral side, multiple metrics also showed between-group differences, but differences between shorter and longer duration groups were not as consistent as on the ipsilateral side. Contralateral hippocampal total volume (P_FDR_ = 0.054), gyrification index (P_FDR_ = 0.299), and dentate gyrus volume (P_FDR_ = 0.990) showed no statistical differences between the two patient groups. Nevertheless, contralateral cortical thickness and subiculum volume in the longer duration group remained significantly lower than in the shorter duration group (P_FDR_ = 0.029 and P_FDR_ = 0.014, respectively). To further validate the robustness of duration-based group analysis, we repeated the duration-stratified analysis after excluding patients with MRI-diagnosed hippocampal sclerosis (see Supplementary Methods S7 and Supplementary Table S11).

**Table 5:** Comparison of hippocampal Z-score metrics between epilepsy patients with shorter and longer disease duration, with pairwise comparisons to controls

Metric	Shorter Duration Mean (SD)	Longer Duration Mean (SD)	p-value (ANOVA)	P Control vs Shorter	P Control vs Longer	P Shorter vs Longer
**Total Hippocampus Metrics**						
Ipsi volume	−1.02 (1.85)	−1.68 (1.76)	<0.001**	<0.001**	<0.001**	<0.001**
Contra volume	−0.51 (1.57)	−0.30 (1.13)	<0.001**	<0.001**	<0.001**	0.054
Ipsi thickness	−0.40 (1.52)	−1.35 (1.80)	<0.001**	<0.001**	<0.001**	<0.001**
Contra thickness	−0.16 (1.33)	−0.39 (1.24)	<0.001**	0.057	<0.001**	0.029*
Ipsi gyrification	−0.96 (1.95)	−1.92 (2.15)	<0.001**	<0.001**	<0.001**	<0.001**
Contra gyrification	−0.33 (1.52)	−0.21 (1.26)	0.001*	<0.001**	0.012*	0.299
Ipsi mean curvature	0.70 (1.81)	1.56 (2.63)	<0.001**	<0.001**	<0.001**	<0.001**
Contra mean curvature	0.29 (1.31)	0.10 (1.31)	0.003*	<0.001**	0.271	0.059
Ipsi intrinsic curvature	1.01 (2.09)	2.08 (3.10)	<0.001**	<0.001**	<0.001**	<0.001**
Contra intrinsic curvature	0.55 (1.94)	0.27 (1.76)	<0.001**	<0.001**	0.012*	0.062
**Subfield Volumes**						
Ipsi subiculum	−0.73 (1.44)	−1.46 (1.47)	<0.001**	<0.001**	<0.001**	<0.001**
Contra subiculum	−0.22 (1.25)	−0.48 (1.34)	<0.001**	0.009*	<0.001**	0.014*
Ipsi CA1	−1.04 (1.78)	−1.91 (2.07)	<0.001**	<0.001**	<0.001**	<0.001**
Contra CA1	−0.52 (1.41)	−0.27 (1.33)	<0.001**	<0.001**	0.002*	0.024*
Ipsi CA2	−0.80 (1.84)	−1.19 (2.17)	<0.001**	<0.001**	<0.001**	0.018*
Contra CA2	−0.75 (1.66)	−0.33 (1.44)	<0.001**	<0.001**	<0.001**	<0.001**
Ipsi CA3	−0.74 (1.78)	−1.60 (2.19)	<0.001**	<0.001**	<0.001**	<0.001**
Contra CA3	−0.42 (1.52)	−0.26 (1.56)	<0.001**	<0.001**	0.008*	0.222
Ipsi CA4	−0.20 (1.61)	−1.08 (1.84)	<0.001**	0.047*	<0.001**	<0.001**
Contra CA4	0.20 (1.39)	0.10 (1.42)	0.101	0.030*	0.276	0.368
Ipsi DG	−0.84 (1.98)	−2.12 (2.46)	<0.001**	<0.001**	<0.001**	<0.001**
Contra DG	−0.22 (1.51)	−0.21 (1.41)	0.029*	0.025*	0.018*	0.990
Ipsi SRLM	−1.00 (1.95)	−2.12 (2.34)	<0.001**	<0.001**	<0.001**	<0.001**
Contra SRLM	−0.37 (1.46)	−0.25 (1.39)	<0.001**	<0.001**	0.006*	0.315

Hippocampal Z-score metrics are presented for epilepsy patients with shorter disease duration (duration < median, *N* = 350) and longer disease duration (duration ≥ median, *N* = 355), where the median disease duration was used as the cut-off for group assignment

Group differences among controls (*N* = 424), shorter duration, and longer duration groups were assessed using one-way ANOVA. Pairwise group comparisons (Control vs. Shorter, Control vs. Longer, Shorter vs. Longer) were performed using Welch’s t-tests, with all *p*-values corrected for multiple comparisons using the Benjamini-Hochberg FDR method

*p < 0.05, **p < 0.001

Ipsi, ipsilateral; Contra, contralateral; CA, cornu ammonis subfield; DG, dentate gyrus; SRLM, stratum radiatum/lacunosum-moleculare

### Longitudinal changes in hippocampal structure

**Fig. 3:**
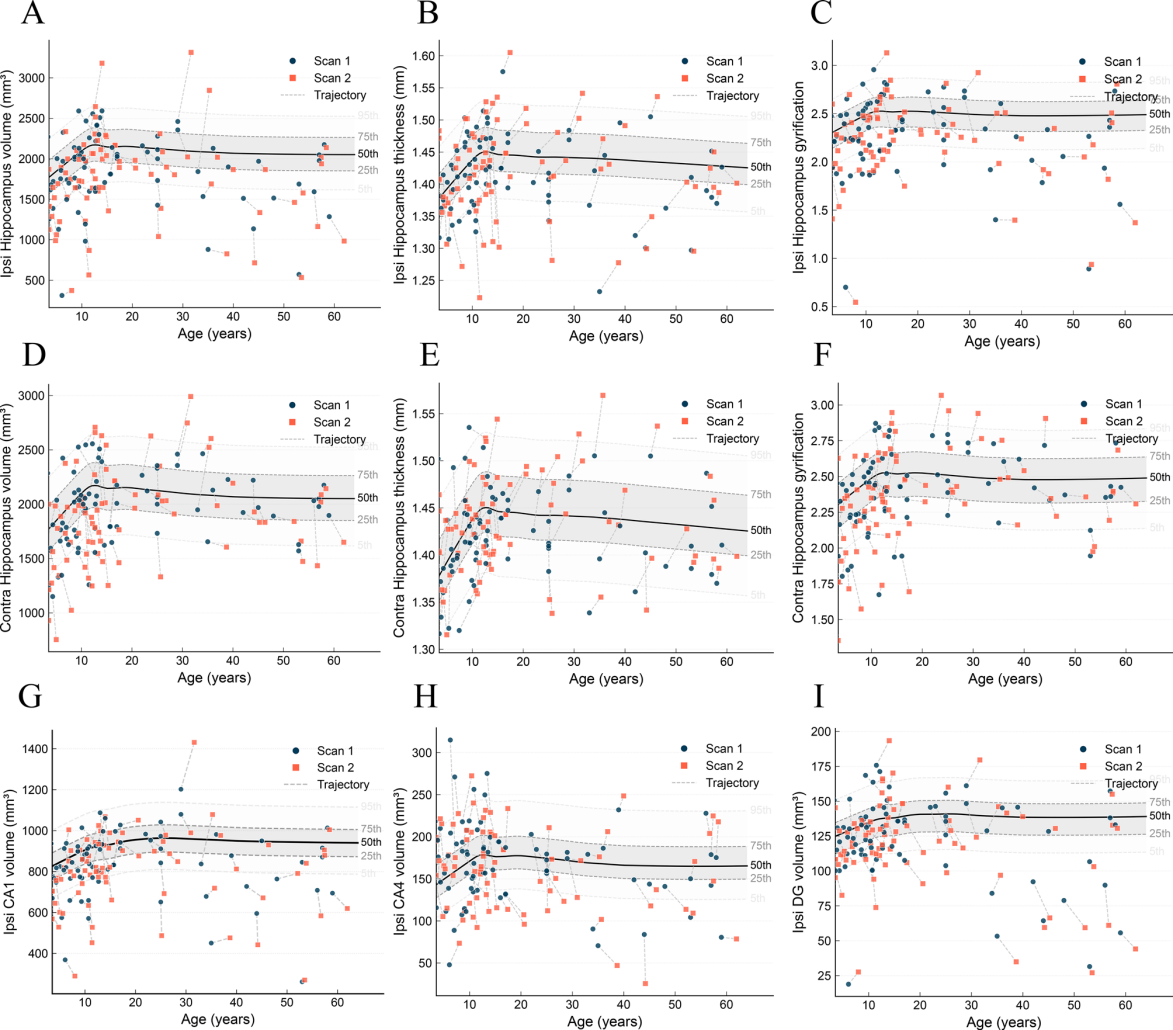
Longitudinal trajectories of selected hippocampal features relative to normative aging. Each panel presents the longitudinal trajectories for selected hippocampal structural metrics. The background normative curves, derived from the healthy control cohort, illustrate the expected age-related distribution. The solid black line represents the median (50th percentile) fitted using LOWESS, with the gray shaded area showing the 95% confidence interval. The dashed lines separately mark the 25th and 75th percentiles. Notably, the normative curves for volume and thickness demonstrate a slight decline with age, reflecting normal aging processes. Colored points mark each patient’s scan 1 and scan 2, connected by dashed lines to indicate within-subject change. Panels display ipsilateral hippocampal volume (**A**), thickness (**B**), and gyrification (**C**); contralateral volume (**D**), thickness (**E**), and gyrification (**F**); and ipsilateral subfields CA1 (**G**), CA4 (**H**), and DG (**I**). Complete trajectories for all hippocampal features are provided in Supplementary Figure S4. All volume metrics are in mm^3^ . Hipp, hippocampal; CA, cornu ammonis subfield; DG, dentate gyrus; SRLM, stratum radiatum/lacunosum-moleculare; LOWESS, locally weighted scatterplot smoothing; IQR, interquartile range

Before conducting longitudinal analysis, we compared baseline characteristics between patients with serial MRI (*N* = 80) and those with a single scan (*N* = 625) to evaluate the representativeness of the longitudinal sub-cohort. The longitudinal subgroup was younger and had shorter disease duration at baseline, whereas sex distribution, age at onset, and seizure-focus localization were comparable (Supplementary Table S12). Over a mean follow-up period of 1.36 ± 1.06 years (Supplementary Figure S5A), linear mixed-effects models revealed significant progressive deviations from age-matched healthy norms in multiple hippocampal structural features (Table 6). Figure 3 visualizes these individual trajectories for key structural metrics against the normative reference curves derived from healthy controls. As shown by the normative percentile bands in Fig. 3, hippocampal volume in healthy individuals exhibits only a modest age-related decline across the sampled age range, whereas patient measurements and longitudinal trajectories frequently deviate substantially from the normative range, supporting that the observed abnormalities are unlikely to be explained by normal aging alone.

**Table 6:** Longitudinal changes in hippocampal Z-Scores based on linear mixed-effects models (*N* = 80)

Variable	Annual Change (per year)	Annual Change 95% CI	Percent Decreased (%)	Adjusted p value
**Total Hippocampus Metrics**				
Ipsi Volume	−0.21	[−0.39, −0.03]	76.2%	0.042*
Contra Volume	−0.13	[−0.28, 0.01]	72.5%	0.135
Ipsi Thickness	0.07	[−0.05, 0.20]	43.8%	0.303
Contra Thickness	0.16	[0.08, 0.25]	32.5%	0.002*
Ipsi Gyrification	−0.16	[−0.24, −0.08]	51.2%	<0.001**
Contra Gyrification	−0.10	[−0.18, −0.03]	66.2%	0.024*
Ipsi Mean Curvature	−0.08	[−0.21, 0.05]	58.8%	0.301
Contra Mean Curvature	−0.09	[−0.19, 0.01]	55.0%	0.138
Ipsi Intrinsic Curvature	0.21	[−0.03, 0.45]	28.7%	0.138
Contra Intrinsic Curvature	0.11	[−0.06, 0.27]	52.5%	0.288
**Subfield Volumes**				
Ipsi Subiculum	−0.03	[−0.16, 0.10]	61.3%	0.778
Contra Subiculum	−0.22	[−0.35, −0.09]	67.5%	0.005*
Ipsi CA1	−0.09	[−0.20, 0.02]	68.8%	0.176
Contra CA1	−0.02	[−0.13, 0.09]	53.8%	0.786
Ipsi CA2	−0.33	[−0.57, −0.08]	53.8%	0.027*
Contra CA2	0.15	[0.00, 0.29]	46.2%	0.087
Ipsi CA3	−0.07	[−0.26, 0.12]	62.5%	0.562
Contra CA3	−0.00	[−0.13, 0.13]	58.8%	0.996
Ipsi CA4	−0.25	[−0.39, −0.11]	52.5%	0.002*
Contra CA4	0.03	[−0.11, 0.16]	53.8%	0.786
Ipsi DG	−0.26	[−0.35, −0.16]	65.0%	<0.001**
Contra DG	−0.18	[−0.27, −0.09]	65.0%	0.001*
Ipsi SRLM	−0.11	[−0.20, −0.02]	66.2%	0.032*
Contra SRLM	−0.14	[−0.24, −0.03]	63.7%	0.030*

Annual rates of change in hippocampal feature Z-scores were estimated using linear mixed-effects models in 80 patients with unilateral focal epilepsy who underwent two MRI scans. The follow-up interval in years between scans was modeled as the primary fixed effect (Time Point), with model selection performed individually for each feature based on Akaike Information Criterion and likelihood ratio tests. Values are expressed as the estimated annual change (per year) in Z-score, with 95% confidence intervals (CI), percentage of patients showing a decrease between scans, and false discovery rate (FDR)-corrected Q-values. Negative annual change indicates progressive deviation below age-matched healthy norms

* *p* < 0.05, ** *p* < 0.001 after Benjamini–Hochberg FDR correction

CI, confidence interval; FDR, false discovery rate; Ipsi, ipsilateral; Contra, contralateral; CA, cornu ammonis subfield; DG, dentate gyrus; SRLM, stratum radiatum/lacunosum-moleculare

At the whole-structure level, ipsilateral hippocampal volume declined by an average of −0.21 Z-score units/year (P_FDR_ = 0.042), with 76.2% of patients showing volume reduction. Ipsilateral gyrification decreased by −0.16/year (P_FDR_ < 0.001) and contralateral gyrification by −0.10/year (P_FDR_ = 0.024, 66.2% of patients affected). Contralateral cortical thickness unexpectedly showed a small but significant increase (+0.16/year, P_FDR_ = 0.002) (Supplementary Figure S5B–D); however, because this was an isolated finding without concordant increases in contralateral hippocampal volume or most contralateral subfield volumes, it should be interpreted cautiously. Although 76.2% of patients showed ipsilateral hippocampal volume reduction, the magnitude of change varied across individuals. We therefore summarized individual annualized Z-score changes using median and interquartile range (IQR) and performed an exploratory comparison by drug-resistant epilepsy status (Supplementary Table S13).

At the subfield level, significant annual volume losses were observed in ipsilateral CA2 (−0.33/year, P_FDR_ = 0.027), CA4 (−0.25/year, P_FDR_ = 0.002), dentate gyrus (−0.26/year, P_FDR_ < 0.001, affecting 65.0% of patients), and SRLM (−0.11/year, P_FDR_ = 0.032). Contralateral subiculum (−0.22/year, P_FDR_ = 0.005, 67.5% affected), dentate gyrus (−0.18/year, P_FDR_ = 0.001, 65.0% affected), and SRLM (−0.14/year, P_FDR_ = 0.030, 63.7% affected) also showed significant progressive atrophy. Other metrics, including mean curvature and intrinsic curvature, did not show statistically significant longitudinal trends after FDR correction. An initial paired t-test analysis is provided in Supplementary Table S14 for reference.

### Sensitivity analyses

To validate the robustness of our main findings, we conducted systematic sensitivity analyses. First, after excluding structural lesions other than hippocampal sclerosis (*n* = 503), the hippocampal morphological differences between epilepsy patients and HCs remained significant, and the correlation between disease duration and hippocampal atrophy was further strengthened (see Supplementary Tables S15, S16, S18, S20, S22). Duration-stratified analysis showed that the longer duration group exhibited more severe hippocampal atrophy compared to the shorter duration group. In MRI-negative patients with complete exclusion of all structural abnormalities (*n* = 313), hippocampal morphological changes, though somewhat attenuated, remained present, particularly evident in key subfields including the subiculum, dentate gyrus, and SRLM (see Supplementary Tables S17, S19, S21, S23). These sensitivity analyses consistently demonstrated that duration-dependent associations are independent of the presence of other structural lesions. Additional syndrome-stratified analyses showed that the main ipsilateral duration-related pattern was observed in both the TLE and non-TLE subgroups. In both groups, longer disease duration was associated with lower ipsilateral hippocampal volume, thickness, and gyrification, together with higher ipsilateral curvature measures, whereas contralateral associations were weaker and less consistent (Supplementary Table S24). These findings indicate that the duration-related hippocampal abnormalities were not restricted to TLE.

To further clarify the interpretability of key subgroup findings, we additionally calculated post-hoc power and minimum detectable effect (MDE) for the primary cross-sectional endpoint (duration–ipsilateral hippocampal volume association) in three prespecified clinically relevant subgroups: patients without MRI-confirmed hippocampal sclerosis (non-HS), MRI-negative patients, and pediatric patients (<18 years) (Supplementary Methods S6; Supplementary Table S25). For the observed effect sizes, the non-HS subgroup remained adequately powered (achieved power = 0.824; 80% power MDE |ρ| = 0.126), whereas the MRI-negative subgroup showed only moderate power (achieved power = 0.684; MDE |ρ| = 0.158). In contrast, the pediatric subgroup was underpowered for small effects (achieved power = 0.134; MDE |ρ| = 0.174). Accordingly, the absence of a significant duration–ipsilateral hippocampal volume association in pediatric patients should be interpreted cautiously as inconclusive rather than as evidence of no association. Taken together, these sensitivity analyses support the robustness of the main duration-related findings after accounting for lesion-related and VPA-related confounding, while also clarifying the limited interpretability of null findings in smaller subgroups.

## Discussion

In this multicenter cohort study, patients with unilateral focal epilepsy showed marked ipsilateral and milder contralateral hippocampal abnormalities, with the most prominent subfield involvement in CA1 and the dentate gyrus. Cross-sectional analyses demonstrated that longer disease duration was associated with greater deviation from healthy hippocampal norms. The syndrome-stratified analysis further suggests that the observed duration-related hippocampal abnormalities are not solely driven by TLE. In non-TLE, hippocampal changes may reflect secondary degeneration within distributed epileptic networks or repeated propagation of epileptic activity to mesial temporal structures, rather than primary mesial temporal pathology [30, 31]. Longitudinal analyses further suggested progressive ipsilateral atrophy in a substantial subset of patients. Together, these findings support a duration-related pattern of hippocampal remodeling and may help explain the higher frequency of HS in adult than pediatric surgical cohorts.

Previous studies have demonstrated associations between disease duration and hippocampal atrophy, but many were constrained by small sample sizes, narrow age ranges, or limited follow-up, which restricted the generalizability of their findings [13, 14]. The ENIGMA study confirmed a negative correlation between disease duration and hippocampal volume but primarily observed changes in the ipsilateral hippocampus [17]. Our cross-sectional analysis yielded a Spearman correlation coefficient of ρ = −0.181 (P_FDR_ < 0.001) between disease duration and ipsilateral hippocampal volume. This effect size is more conservative than the pooled effect size reported in a comprehensive meta-analysis of drug-resistant TLE (*r* = −0.42) [32]. This discrepancy likely reflects differences in cohort composition and methodology. First, whereas many prior studies predominantly focused on drug-resistant TLE, our multicenter cohort included unilateral focal epilepsy with diverse seizure localizations. Second, we applied an age-adjusted normative-modeling framework using Z-scores; because normal aging itself contributes to hippocampal volume loss, differences in how age-related effects were modeled across studies may also have contributed to a stronger apparent association with disease duration in earlier reports. At the same time, the observed cross-sectional effect size remains small, indicating that disease duration captures only a limited portion of between-patient variability in hippocampal structure. Accordingly, disease duration may be better interpreted as a contextual or group-level disease marker rather than a stand-alone individualized predictor of hippocampal structural abnormality. In the longitudinal analysis, ipsilateral hippocampal volume declined by −0.21 Z-score units/year, with 76.2% of patients showing volume reduction. Although direct conversion between annual percentage volume loss and annual Z-score change is not straightforward, this rate is directionally consistent and of a comparable order to prior longitudinal volumetric studies in temporal lobe epilepsy reporting progressive ipsilateral hippocampal atrophy over time [33, 34]. Taken together, these findings support a cumulative atrophy model, while also indicating that the apparent rate of progression is likely to vary according to epilepsy syndrome, disease severity, and cohort selection. In addition, because serial MRI in our study was clinically indicated rather than protocol-mandated, the longitudinal subgroup may have been enriched for patients with changing disease status, which could bias estimated progression rates upward.

Our neuroimaging findings are consistent with histopathological evidence showing decreased neuronal density in the ipsilateral hippocampus with longer disease duration [35]. Notably, our subfield analysis identified CA1 and the dentate gyrus as the most prominent duration-associated “hotspot” regions. This pattern parallels the well-described distribution of neuronal loss and gliosis in hippocampal sclerosis [11]. Experimental models, such as the classic kindling paradigm, further demonstrated that repeated seizures can induce progressive, region-specific neuronal loss in the hippocampus [8, 36–38]. At the molecular level, cumulative damage may be mediated by several pathways, including excitotoxicity from excessive glutamate release, seizure-induced inflammatory cascades, and maladaptive signaling such as mTORC1 activation [39–42]. CA1 pyramidal neurons appear particularly susceptible to glutamate-driven excitotoxicity and metabolic/oxidative stress, which may help explain the disproportionate CA1 involvement under recurrent seizure burden [43, 44]. In the DG–hilar circuitry, mossy cells are glutamatergic and highly excitable, and experimental work supports their vulnerability under sustained hyperexcitability, providing a plausible circuit-level contributor to DG structural change [41]. In addition, a recent study integrating histology with microelectrode array recordings indicates that a chronically hyperexcitable microenvironment may suppress DG neurogenesis and/or survival [45]. Collectively, these subregion-specific vulnerabilities provide mechanistic context for the pronounced duration-associated atrophy observed in CA1 and DG in our cohort.

However, it must be acknowledged that approximately 40% of patients in our longitudinal cohort maintained relatively stable hippocampal structure, indicating that the ‘duration-related structural alteration’ hypothesis does not apply to all epilepsy patients. This neuroimaging observation is echoed at the pathological level: an autopsy analysis of long-term epilepsy patients confirmed the existence of a distinct subgroup that, despite experiencing decades of severe seizures, showed no significant hippocampal neuronal loss [46]. The “progressive” versus “non-progressive” dichotomy we observed may reflect complex interactions among multiple factors including duration-dependent accumulation, initial injury severity, overall epileptic activity, and individual genetic susceptibility.

A recent twin study of 80 pairs with temporal lobe epilepsy provides insight into this heterogeneity. It demonstrated a substantial germline genetic contribution overall, but the signal was driven primarily by non-lesional MTLE without hippocampal sclerosis; by contrast, concordance for MTLE-HS among monozygotic pairs was rare and largely confined to specific contexts such as neurofibromatosis type 1 (NF1), suggesting that cases driven solely by germline genetics are relatively uncommon [47]. This implies that the development of HS often requires factors in addition to genetic susceptibility. Initial brain injury, particularly complex febrile seizures in childhood, is considered another critical initiating factor [48–52]. The FEBSTAT study found that children with complex febrile seizures and acute-phase hippocampal MRI abnormalities carry a markedly elevated risk of HS [48, 49, 53]. Animal models provide mechanistic support for this “initial injury” concept, showing that severe initial limbic insults can produce widespread hippocampal neuronal loss, whereas kindling models (without an initial injury) do not exhibit comparable structural abnormalities [8, 52].

Taken together, we propose a two-stage, multiple-hit model: initiating factors—whether genetic susceptibility or early-life hippocampal injury—establish a vulnerable substrate, upon which a ‘second hit’ of cumulative injury after epilepsy onset may subsequently contribute to progressive remodeling and neuronal loss toward an HS-like pattern. The severity of this cumulative damage is further modulated by factors like seizure burden and can manifest as pathological remodeling, such as mossy fiber sprouting [8, 52]. Consistent with this, previous studies have reported that patients with mesial temporal lobe epilepsy who have more frequent seizures and longer disease duration exhibit more severe hippocampal atrophy [33]. All of these collectively explain the coexistence of “progressive” and “non-progressive” patient phenotypes and suggest that future research should incorporate multi-omics factors for stratified studies to provide theoretical foundations for individualized prevention and intervention.

Beyond the aforementioned disease-related factors, our supplementary analysis confirmed an independent atrophic effect of valproic acid on hippocampal structure, which is highly consistent with multiple recent studies. Pardoe et al. found that valproic acid use was associated with bilateral hippocampal atrophy, with effects exceeding those of epilepsy itself on hippocampal volume [29]. Prospective studies further demonstrated that valproic acid can accelerate hippocampal volume loss [54]. Notably, valproic acid-related hippocampal atrophy presents a relatively bilaterally symmetric pattern, while the disease duration-related atrophy found in this study shows ipsilateral predominance. Additionally, the former may be related to osmotic effects and exhibits some reversibility [25, 27, 54], whereas the latter may reflect irreversible neuronal loss and gliosis. Therefore, our sensitivity analysis suggests that VPA alone is unlikely to fully account for the observed duration-related pattern. Nevertheless, residual confounding from other anti-seizure medications cannot be excluded, and the present findings should not be interpreted as demonstrating complete medication independence.

From a translational perspective, our data supports a practical imaging-surveillance framework rather than a fixed clinical threshold. Among the evaluated metrics, ipsilateral total hippocampal volume appears to be the most pragmatic primary marker for serial assessment because it showed the most consistent abnormalities across the cross-sectional, duration-stratified, and longitudinal analyses [18]. Dentate gyrus volume may serve as a secondary subfield marker when reliable subfield segmentation is available, whereas ipsilateral CA1 may be more informative as a complementary marker of regional vulnerability than as a stand-alone serial endpoint. By contrast, contralateral thickness and curvature metrics may be less suitable as primary monitoring indicators because their associations were smaller and their longitudinal behavior was less consistent in our dataset. From a management perspective, these findings do not establish a fixed operative threshold, do not justify a uniform MRI follow-up interval, and do not support the use of any single hippocampal metric as an isolated trigger for treatment escalation. Instead, quantitative hippocampal MRI may be most useful as an adjunct to standard multimodal assessment by helping contextualize subtle hippocampal abnormalities, support risk stratification, and reinforce timely referral for comprehensive presurgical evaluation once drug resistance is suspected or established. Importantly, the median duration split used in this study (11 years) was intended for descriptive stratification rather than as an intervention threshold. Previous research using a comparable normative modeling framework showed that more than 90% of hippocampal volumes in patients with histologically confirmed hippocampal sclerosis fall below the 5th percentile of the healthy population (approximately Z = −1.65) [18]. Based on the observed annual decline in ipsilateral hippocampal volume in our longitudinal cohort (−0.21 Z-score units/year), a simplified illustrative model suggests that a patient beginning near the normative mean at seizure onset could approach the lower normative tail within roughly the first decade of disease, assuming approximate linear decline. This estimate should be interpreted as hypothesis-generating rather than prescriptive because it assumes a normative baseline at seizure onset, approximate linearity of decline, and no major distortion by unmeasured clinical factors. In pediatric patients, earlier consideration of baseline quantitative MRI may be reasonable given ongoing neurodevelopment and longer potential lifetime exposure to epilepsy, but our data are insufficient to define separate evidence-based surveillance schedules for children versus adults. Likewise, although the present findings are compatible with the concept of an earlier disease phase during which structural deviation may become detectable, they do not identify an evidence-based therapeutic cutoff for interventions targeting excitotoxic or inflammatory pathways [39, 55]. A more appropriate translational implication of the current data is to inform future prospective studies: such studies should enroll patients early in the disease course, apply standardized serial epilepsy MRI with consistent high-resolution 3D T1-weighted acquisition together with routine T2/FLAIR, and test whether earlier optimization of seizure control or mechanism-informed adjunctive interventions can attenuate ipsilateral hippocampal decline and improve long-term outcomes.

This study has several limitations. First, our findings must be interpreted within the inherent limitations of neuroimaging. MRI provides an indirect, macroscopic view of brain structure, and the progressive changes we observed are the net result of a complex underlying biology. While we demonstrate a strong association between disease duration and atrophy, it remains unclear whether these changes are a direct consequence of clinical seizures (ictal events), the cumulative effect of subclinical interictal discharges, or other unmeasured disease-related processes, such as neuroinflammatory processes. Reverse causation also cannot be excluded; whereby early subtle hippocampal pathology may independently contribute to both prolonged disease duration and more severe structural abnormalities.

Second, the retrospective design and cohort heterogeneity limit our ability to precisely quantify variables like seizure burden. Seizure frequency and clinical semiology were not prospectively and uniformly captured across this retrospective multicenter cohort. Because both are time-varying clinical features that may change substantially with treatment response, remission, and disease evolution, a single retrospectively extracted value would not provide a stable or biologically meaningful representation of cumulative seizure burden. Accordingly, our duration-related findings should not be interpreted as fully independent of cumulative seizure load. To partially address clinical severity, we performed an exploratory stratified analysis using drug-resistant epilepsy (DRE) status as a pragmatic surrogate of disease severity/clinical progression. The overall pattern of annualized hippocampal change was broadly similar between DRE and non-DRE subgroups. However, DRE does not substitute for direct longitudinal quantification of seizure burden. Another limitation concerns anti-seizure medication exposure. Although we performed a targeted sensitivity analysis for valproate, detailed information on dosage, cumulative exposure, and longitudinal treatment history was not consistently available, precluding reliable dose-response modeling. This limitation is particularly relevant in a cross-age cohort including pediatric patients, in whom clinically meaningful dose comparisons would require weight-adjusted exposure measures. Accordingly, the valproate findings should be interpreted as sensitivity data addressing potential confounding rather than as evidence of a medication dose-response effect. Other anti-seizure medication classes were not modeled individually because reliable harmonization of class-specific lifetime exposure, cumulative dose, regimen switching, and polytherapy patterns were not feasible in this retrospective multicenter dataset. Third, the longitudinal sub-cohort was not assembled through a standardized prospective protocol; instead, repeat MRI scans were obtained as part of routine clinical care. This may introduce indication bias, as patients undergoing repeat imaging could have had more active or evolving diseases. In addition, the unexpected increase in contralateral thickness should be interpreted cautiously rather than as definitive evidence of progressive contralateral hypertrophy. Although limited compensatory or developmental remodeling cannot be excluded, this finding was not accompanied by concordant contralateral volumetric increases. Methodological factors may also have contributed, including physiological between-scan variability (e.g., hydration state) and residual instability of automated thickness estimation despite standardized 3D T1 acquisition, site adjustment, and multi-step quality control. Conversely, the relatively short follow-up period may have been insufficient to capture slower, long-term structural changes. Future prospective studies with standardized imaging intervals and systematic retention tracking are needed to provide unbiased estimates of hippocampal progression rates. Another technical limitation is that automated hippocampal subfield segmentation remains method dependent. Although all scans were processed using a single harmonized pipeline and underwent multi-step quality control, subfield boundaries and absolute morphometric estimates may vary across segmentation software, atlas definitions, and preprocessing strategies, particularly for smaller subfields. Therefore, our findings are best interpreted as internally consistent within-pipeline comparisons rather than as directly interchangeable absolute measurements across analytical platforms. Moreover, despite controlling age in our normative models, the inherent high collinearity between age and disease duration makes it difficult to completely disentangle their independent effects. A further methodological consideration is that bilateral hippocampi from the same healthy control are partially correlated. Therefore, using both hemispheres to construct the normative reference distribution may modestly inflate the effective reference sample size. Although our main inferential analyses were conducted at the patient level after lateralization, future studies should further evaluate hemisphere-specific normative models or mixed-effects normative frameworks with subject-level random effects. Finally, although the multicenter design improved internal robustness, all participants were recruited from three tertiary medical centers in western China. Regional differences in referral patterns, case mix, ethnicity, treatment access, and healthcare context may limit the generalizability of these findings to other populations and health systems. External validation in geographically broader and ethnically diverse cohorts will therefore be important. These limitations also define specific priorities for future work. Prospective multicenter cohorts with standardized longitudinal capture of seizure frequency, seizure burden, epilepsy syndrome classification, history of status epilepticus, and weight-adjusted cumulative anti-seizure medication exposure will be necessary to disentangle the independent contributions of clinical severity, syndrome heterogeneity, and medication-related effects. Pre-specified longitudinal imaging protocols with fixed follow-up intervals and systematic retention tracking will be important to reduce indication bias and provide less biased estimates of hippocampal progression rates. To better distinguish developmental or aging effects from disease-duration effects, future studies should incorporate onset-anchored prospective designs, narrower age-band recruitment, or parallel pediatric and adult cohorts. In addition, cross-platform benchmarking studies integrating multiple segmentation pipelines, higher-resolution multimodal MRI, and expert/manual validation subsets will be important to strengthen subfield-level reproducibility. Despite these limitations, this study, through its large-scale, multicenter, cross-age design combined with longitudinal follow-up and advanced neuroimaging analysis methods, provides new perspectives for understanding disease duration and hippocampal morphological changes. Future prospective cohort studies, multi-omics-based stratified analyses, and longer-term longitudinal follow-up will help further validate and refine these findings.

## Electronic supplementary material

Below is the link to the electronic supplementary material.

Supplementary Material 1

Supplementary Material 2

Supplementary Material 3

Supplementary Material 4

Supplementary Material 5

Supplementary Material 6

## Data Availability

The raw imaging and clinical data are protected and cannot be made publicly available due to data privacy laws. If you need to access the raw data, you can submit a request to the corresponding author. The local ethics committee will promptly review the request and make a decision.
