# Supplementary material for: Duration-dependent hippocampal structural changes in focal epilepsy: multicenter neuroimaging evidence
Source: J Transl Med. 2026 May 8;24:843. doi: 10.1186/s12967-026-08230-x (PMC13326556; doi:10.1186/s12967-026-08230-x)
Supplement: Supplementary file 1 — Supplementary Material 1 [file 12967_2026_8230_MOESM1_ESM.docx]

**Supplementary Methods S1: MRI Acquisition, Parameters, and Quality Control**

Brain magnetic resonance imaging (MRI) data for all study subjects were acquired on 3.0T scanners. A standardized high-resolution isotropic protocol (1 × 1 × 1 mm voxels) using 3D T1-weighted sequences was employed across all sites. All MRI scans were performed by uniformly trained technicians following standardized protocols.

The specific scanner models and acquisition parameters for each center were as follows: Chongqing Site (Children's Hospital of Chongqing Medical University): Imaging was performed on a Philips Achieva 3.0T scanner with the following parameters: Repetition Time (TR) = 2000 ms; Echo Time (TE) = 20 ms; Inversion Time (TI) = 800 ms. Lanzhou Site (Second Hospital of Lanzhou University): A Philips Achieva 3.0T scanner was used with parameters set to TR = 2500 ms, TE = 25 ms, and TI = 900 ms. Xinjiang Site (Second Affiliated Hospital of Xinjiang Medical University): A Philips Ingenia CX 3.0T scanner was used with parameters of TR = 2100 ms, TE = 30 ms, and TI = 900 ms. To minimize motion artifacts, foam padding was used for pediatric subjects during the scans. Following acquisition, all raw MRI images underwent a rigorous quality control process, conducted independently by two experienced researchers. This procedure ensured that all images had whole-brain coverage, were free of significant artifacts, and possessed clear structural detail suitable for analysis. To further control for potential multicenter heterogeneity in the data, the scanning site was included as a covariate in all subsequent statistical analyses.

To ensure that clinical diagnostic criteria for hippocampal sclerosis (HS) were applied consistently across the three participating centers, we performed a validation analysis using surgical histopathology as the gold standard. Among the enrolled cohort, a subset of 277 patients underwent surgical resection and had available hippocampal resection specimens for histopathological evaluation. We compared the pre-surgical visual MRI diagnosis (HS vs. non-HS) with the post-surgical pathological outcome for each center independently. The concordance was assessed using Cohen’s Kappa coefficient, overall accuracy, and sensitivity. All three centers demonstrated high diagnostic accuracy and substantial agreement with pathological findings: Chongqing Site (CQ): In 35 patients with hippocampal resection specimens, the MRI diagnosis achieved a Cohen’s Kappa of 0.750, with an accuracy of 88.6%. Xinjiang Site (XJ): In 79 patients with hippocampal resection specimens, the MRI diagnosis achieved a Cohen’s Kappa of 0.725, with an accuracy of 92.4% and a sensitivity of 92.6%. Lanzhou Site (LZ): In 163 patients with hippocampal resection specimens, the MRI diagnosis achieved a Cohen’s Kappa of 0.610, with an accuracy of 90.2% and a sensitivity of 92.3%.

**Supplementary Methods S2: Detailed Segmentation and Feature Extraction**

MRI images from all participants were first processed using FreeSurfer software (version 7.4; <http://surfer.nmr.mgh.harvard.edu/>) to estimate total intracranial volume (TIV).21 Subsequently, the open-source neuroimaging analysis tool HippUnfold (version 1.5.2, <https://github.com/khanlab/hippunfold>) was used for automated hippocampal segmentation and surface reconstruction,19 partitioning bilateral hippocampi into subfields including subiculum, CA1, CA2, CA3, CA4, dentate gyrus (DG), SRLM, and cyst regions, with extraction of subfield volumes (Figure 2). The automated interpretable detection tool AID-HS (version 1.0.0, <https://github.com/MELDProject/AID-HS>) was then employed to extract global surface morphological features of bilateral hippocampi,18 including total hippocampal volume, cortical thickness, gyrification index, mean curvature, and intrinsic curvature. The AID-HS classifier achieved a detection rate of 90.1% for unilateral HS patients and 79.2% for MRI-negative but histopathologically confirmed HS cases.18

All segmentation results were initially screened using the "Quality check of segmentation" scores output by AID-HS, with segmentations scoring below 0.7 excluded. The remaining data underwent independent visual inspection by two experienced neuroradiologists, blinded to clinical information, and segmentations that clearly deviated from actual hippocampal anatomy were excluded. Each rater classified segmentations as “OK”, “Maybe”, or “Fail”. Because both “Maybe” and “Fail” lead to the same downstream QC action, we collapsed them into a single “needs adjudication” category for inter-rater reliability analyses. Inter-observer agreement was substantial (Cohen’s κ = 0.789). In cases of disagreement between the two reviewers, final adjudication was performed by a senior neuroradiologist. Agreement with the adjudicated final decision was 95.2% for Reviewer 1 and 98.1% for Reviewer 2).

**Supplementary Methods S3: Construction of Normative Reference Curves for Longitudinal Visualization**

To provide a normative context for visualizing individual patient trajectories, we generated age-based reference curves for each hippocampal feature using data from the entire healthy control cohort. Specifically, for each metric, we employed locally weighted scatterplot smoothing (LOWESS) with a smoothing parameter of 0.5 to model the relationship between age and the hippocampal feature in healthy individuals. Based on this non-parametric regression, we calculated and plotted the 5th, 25th, 50th (median), 75th, and 95th percentile curves. These curves represent the expected distribution of each feature across the lifespan in a healthy population. These reference curves were then used to overlay individual patient trajectories, with Scan 1 and Scan 2 connected by dashed lines to illustrate longitudinal change relative to the normative range.

**Supplementary Methods S4: Age–Duration Collinearity and Interaction Assessment**

Although our primary imaging outcomes were standardized using age-based normative models (including age, sex, TIV, and site covariates), the inherent correlation between patient age and epilepsy duration cannot be fully eliminated. We performed additional analyses in the patient cohort to explicitly evaluate age–duration dependence and potential effect modification.

Collinearity assessment: We quantified the association between age and disease duration using Spearman’s rank correlation and calculated variance inflation factors (VIF) from a linear model including both variables. Age and duration were strongly correlated (Spearman ρ = 0.813); however, the VIF for duration was 3.11, indicating no severe multicollinearity (VIF < 5).

Age-adjusted duration associations: For each hippocampal Z-score metric, we computed (i) the zero-order Spearman correlation with disease duration and (ii) partial Spearman correlations between duration and the metric while controlling for age (rank-based residualization). After adjusting for age, the association between disease duration and ipsilateral hippocampal volume remained statistically significant (partial Spearman ρ = −0.098, P_FDR_ = 0.044), and several subfield measures showed consistent age-independent duration effects (Supplementary Table S8).

Effect-modification analyses: First, we tested a continuous age×duration interaction by fitting linear models of the form Z-score ~ duration + age + duration×age, with age and duration mean-centered. Site and sex were included as covariates (categorical fixed effects), and heteroskedasticity-robust standard errors were used for inference. The age×duration interaction was not significant after FDR correction, indicating no robust evidence that age modifies the duration–hippocampal associations (Supplementary Table S9). Second, we repeated Spearman correlations within pediatric (<18 years) and adult (≥18 years) subgroups. Duration-related associations were robust in adults, whereas no associations survived FDR correction in the pediatric cohort, likely reflecting restricted duration range and limited statistical power (Supplementary Table S8). Notably, 37% of pediatric patients had duration ≤ 1 year, which may limit detection of cumulative structural effects that manifest over longer time scales.

**Supplementary Methods S5: Anti-Seizure Medication Effect Analysis**

To assess potential confounding effects of anti-seizure medications on hippocampal structure, we performed a supplementary analysis focusing on valproate sodium (VPA) usage, given previous reports of VPA-associated brain atrophy. Because comprehensive lifetime ASM exposure (e.g., cumulative dose, regimen changes, and polytherapy over long disease courses) could not be reliably harmonized across this retrospective multicenter cohort, we used VPA as a pragmatic, literature-supported medication proxy for sensitivity testing.

We identified patients with complete medication records documented through multidisciplinary team (MDT) discussions to ensure accurate medication history and avoid potential bias from undocumented medication changes at external centers. From these cases, patients were further categorized based on VPA exposure: VPA users were defined as patients with documented VPA treatment for more than 6 months (N=70), and VPA non-users were defined as patients who had never received VPA treatment (N=134). Patients with short-term VPA exposure (<6 months) were excluded from this analysis to ensure clear group distinction. Due to the retrospective nature of medication history collection and the strict inclusion criteria for medication exposure, sample size was limited. This analysis serves primarily as a sensitivity assessment of potential medication confounding effects. In addition, to directly address potential medication confounding in the primary duration models, we refitted the duration–hippocampal analyses in the same medication-documented subset by including VPA exposure as a covariate. For each hippocampal Z-score metric, we fitted a linear mixed-effects model with duration and VPA as fixed effects and site as a random intercept. Multiple-comparison correction was performed using the Benjamini–Hochberg FDR procedure across all metrics, applied separately for duration and VPA effects (Supplementary Table S4-5).

Statistical comparisons used one-way ANOVA with post hoc Welch's t-tests, with Benjamini-Hochberg FDR correction for multiple comparisons.

**Supplementary Methods S6: Model-consistent post-hoc power and minimum detectable effect (MDE)**

To provide statistical support for sample size, we performed model-consistent sensitivity analyses for two prespecified key outcomes: (i) the cross-sectional association between disease duration and ipsilateral hippocampal volume; and (ii) the longitudinal annual rate of ipsilateral hippocampal volume change.

Power and the minimum detectable correlation were approximated using Fisher’s z transformation (two-sided α=0.05). Achieved power was computed at the observed Spearman correlation, and MDE was defined as the smallest |ρ| yielding 80% power.

Using the same Fisher’s z–based framework, we estimated achieved power and the MDE for the primary cross-sectional endpoint (duration–ipsilateral hippocampal volume association) in three clinically relevant prespecified subgroups: patients without MRI-confirmed hippocampal sclerosis (non-HS), MRI-negative patients, and pediatric patients (<18 years). For each subgroup, achieved power was calculated at the observed Spearman correlation, and MDE was defined as the smallest absolute correlation yielding 80% power. (Supplementary Table S25)

**Supplementary Methods S7: Disease Duration Subgroup Analysis Excluding HS Patients**

To test whether the effect of disease duration on hippocampal structure is independent of hippocampal sclerosis (HS), we repeated the duration-based subgroup analysis in the cohort after excluding all patients with MRI-confirmed HS. Patients were divided into shorter- and longer-duration subgroups using the same median split as in the main analysis, and compared with healthy controls using one-way ANOVA and post hoc Welch’s t-tests, with FDR correction. Both patient subgroups without HS showed significant hippocampal structural deficits compared to controls in most global and subfield metrics. However, the difference between the shorter- and longer-duration groups was not statistically significant (see Supplementary Table S11).

In our study, HS is considered the progressive pathological outcome of chronic epilepsy. By excluding HS patients, especially those in the longer-duration group, we removed cases with the most severe and cumulative structural abnormalities. Thus, the absence of a significant gradient between patient subgroups is expected and supports the concept that the observed duration-dependent gradient in the full cohort is largely driven by the accumulation of HS cases in patients with prolonged disease duration.

**Supplementary Table S1.** **Consistency between MRI-based Diagnosis and Histopathology across Centers**

| Center | N (Surgical) | Cohen’s Kappa | Accuracy | Sensitivity | Specificity |
| --- | --- | --- | --- | --- | --- |
| Chongqing (CQ) | 35 | 0.75 | 88.60% | 71.40% | 100.00% |
| Xinjiang (XJ) | 79 | 0.725 | 92.40% | 92.60% | 90.90% |
| Lanzhou (LZ) | 163 | 0.61 | 90.20% | 92.30% | 76.20% |

Data represents the concordance between pre-surgical MRI diagnosis and post-surgical histopathological findings in a subset of 277 patients with available hippocampal resection specimens. MRI diagnosis was based on visual identification of hippocampal atrophy and T2/FLAIR hyperintensity following ILAE Neuroimaging Task Force recommendations.

Cohen’s Kappa, accuracy, sensitivity, and specificity were calculated for each center independently to verify the consistency of applying MRI diagnostic criteria across centers (center-stratified MRI–histopathology agreement)

CQ, Chongqing; XJ, Xinjiang; LZ, Lanzhou; HS, hippocampal sclerosis.

**Supplementary Table S2. Baseline Characteristics of Patient Cohorts Across Study Sites**

| **Characteristic** | **Site CQ (N=173)** | **Site LZ (N=385)** | **Site XJ**  **(N=147)** | **P value** |
| --- | --- | --- | --- | --- |
| Age (years), mean ± SD | 8.1 ± 3.6 | 31.0 ± 13.6 | 30.8 ± 10.3 | <0.001** |
| Sex, female/male (n) | 94/79 | 200/185 | 85/62 | 0.471 |
| Age at onset (years), mean ± SD | 5.3 ± 3.3 | 11.8 ± 8.8 | 13.4 ± 9.7 | <0.001** |
| Disease duration (years), mean ± SD | 2.8 ± 3.0 | 19.3 ± 14.1 | 17.4 ± 11.4 | <0.001** |
| Lateralization, Left/Right (n) | 78/95 | 209/176 | 86/61 | 0.041* |
| Localization of seizure focus, n (%) |  |  |  | <0.001** |
| FL | 56 (32.4%) | 73 (19.0%) | 38 (25.9%) |  |
| IL | 0 (0.0%) | 3 (0.8%) | 0 (0.0%) |  |
| OL | 10 (5.8%) | 6 (1.6%) | 2 (1.4%) |  |
| PL | 17 (9.8%) | 25 (6.5%) | 7 (4.8%) |  |
| TL | 76 (43.9%) | 267 (69.4%) | 96 (65.3%) |  |
| U | 14 (8.1%) | 11 (2.9%) | 4 (2.7%) |  |

Baseline demographic and clinical characteristics of patients with focal epilepsy across the three study sites: Chongqing (CQ, N=173), Lanzhou (LZ, N=385), and Xinjiang (XJ, N=147).
P-values were calculated using one-way ANOVA for continuous variables and the chi-square test for categorical variables. p < 0.05 considered statistically significant.

* p < 0.05, ** p < 0.001.
CQ, Chongqing; LZ, Lanzhou; XJ, Xinjiang; FL, frontal lobe; IL, insular lobe; OL, occipital lobe; PL, parietal lobe; TL, temporal lobe; U, undetermined.

**Supplementary Table S3. Group Differences in Hippocampal Z-Score Metrics Between Focal Epilepsy Patients and Healthy Controls**

| **Metric** | **Mean (SD)** | **t-statistic** | **Cohen’s d** | **P-value (raw)** | **P value (FDR)** |
| --- | --- | --- | --- | --- | --- |
| **Total Hippocampus Metrics** |  |  |  |  |  |
| Ipsi volume | -1.35 (1.84) | -16.00 | -0.98 | <0.001** | <0.001** |
| Contra volume | -0.41 (1.37) | -5.74 | -0.35 | <0.001** | <0.001** |
| Ipsi thickness | -0.88 (1.73) | -10.82 | -0.66 | <0.001** | <0.001** |
| Contra thickness | -0.28 (1.29) | -4.06 | -0.25 | <0.001** | <0.001** |
| Ipsi gyrification | -1.44 (2.11) | -15.50 | -0.95 | <0.001** | <0.001** |
| Contra gyrification | -0.27 (1.39) | -3.81 | -0.23 | <0.001** | <0.001** |
| Ipsi mean curvature | 1.13 (2.30) | 11.39 | 0.70 | <0.001** | <0.001** |
| Contra mean curvature | 0.19 (1.31) | 2.80 | 0.17 | 0.005* | 0.005* |
| Ipsi intrinsic curvature | 1.55 (2.70) | 13.73 | 0.84 | <0.001** | <0.001** |
| Contra intrinsic curvature | 0.41 (1.85) | 4.78 | 0.29 | <0.001** | <0.001** |
| **Subfield Volumes** |  |  |  |  |  |
| Ipsi subiculum | -1.10 (1.50) | -14.70 | -0.90 | <0.001** | <0.001** |
| Contra subiculum | -0.35 (1.31) | -5.12 | -0.31 | <0.001** | <0.001** |
| Ipsi CA1 | -1.48 (1.98) | -16.64 | -1.02 | <0.001** | <0.001** |
| Contra CA1 | -0.40 (1.38) | -5.59 | -0.34 | <0.001** | <0.001** |
| Ipsi CA2 | -1.00 (2.03) | -11.01 | -0.68 | <0.001** | <0.001** |
| Contra CA2 | -0.54 (1.57) | -7.05 | -0.43 | <0.001** | <0.001** |
| Ipsi CA3 | -1.18 (2.04) | -12.92 | -0.79 | <0.001** | <0.001** |
| Contra CA3 | -0.34 (1.54) | -4.47 | -0.27 | <0.001** | <0.001** |
| Ipsi CA4 | -0.64 (1.78) | -7.76 | -0.48 | <0.001** | <0.001** |
| Contra CA4 | 0.15 (1.40) | 2.04 | 0.13 | 0.042* | 0.042* |
| Ipsi DG | -1.49 (2.32) | -14.87 | -0.91 | <0.001** | <0.001** |
| Contra DG | -0.22 (1.46) | -2.93 | -0.18 | 0.003* | 0.004* |
| Ipsi SRLM | -1.57 (2.23) | -16.14 | -0.99 | <0.001** | <0.001** |
| Contra SRLM | -0.31 (1.43) | -4.24 | -0.26 | <0.001** | <0.001** |

Z-score metrics for hippocampal total and subfield structures comparing focal epilepsy patients (N=705) with healthy controls (N=424). Group comparisons were performed using Welch's independent samples t-test. P values were corrected for multiple comparisons using the Benjamini-Hochberg False Discovery Rate (FDR) method.

* p < 0.05, ** p < 0.001.

Ipsi, ipsilateral; Contra, contralateral; CA, cornu ammonis subfield; DG, dentate gyrus; SRLM, stratum radiatum/lacunosum-moleculare.

**Supplementary Table S4. Hippocampal Z-Score Comparison: Valproate Effect Analysis**

| **Variable** | **Valproate (+) Mean (SD)** | **Valproate (-) Mean (SD)** | **ANOVA P-value** | **P**  **Control vs Valproate (+)** | **P**  **Control vs Valproate (-)** | **P**  **Valproate (+) vs Valproate (-)** |
| --- | --- | --- | --- | --- | --- | --- |
| **Total Hippocampus Metrics** |  |  |  |  |  |  |
| Ipsi volume | -1.42 (1.64) | -0.80 (1.84) | <0.001** | <0.001** | <0.001** | 0.038* |
| Contra volume | -1.16 (1.70) | -0.47 (1.45) | <0.001** | <0.001** | 0.001* | 0.021* |
| Ipsi thickness | -0.50 (1.37) | -0.15 (1.37) | 0.003* | 0.005* | 0.295 | 0.109 |
| Contra thickness | -0.48 (1.22) | 0.06 (1.21) | 0.001* | 0.003* | 0.640 | 0.020* |
| Ipsi gyrification | -1.26 (1.77) | -0.69 (1.87) | <0.001** | <0.001** | <0.001** | 0.076 |
| Contra gyrification | -0.98 (1.66) | -0.28 (1.45) | <0.001** | <0.001** | 0.049* | 0.020* |
| Ipsi mean curvature | 0.81 (1.74) | 0.58 (1.81) | <0.001** | <0.001** | 0.001* | 0.435 |
| Contra mean curvature | 0.72 (1.42) | 0.31 (1.17) | <0.001** | <0.001** | 0.010* | 0.076 |
| Ipsi intrinsic curvature | 1.18 (2.04) | 0.88 (1.88) | <0.001** | <0.001** | <0.001** | 0.364 |
| Contra intrinsic curvature | 1.19 (2.54) | 0.53 (1.55) | <0.001** | <0.001** | <0.001** | 0.076 |
| **Subfield Volumes** |  |  |  |  |  |  |
| Ipsi subiculum | -0.91 (1.20) | -0.39 (1.35) | <0.001** | <0.001** | 0.004* | 0.023* |
| Contra subiculum | -0.49 (1.34) | -0.11 (1.21) | 0.003* | 0.005* | 0.383 | 0.076 |
| Ipsi CA1 | -1.20 (1.42) | -0.77 (1.58) | <0.001** | <0.001** | <0.001** | 0.076 |
| Contra CA1 | -1.11 (1.43) | -0.49 (1.34) | <0.001** | <0.001** | <0.001** | 0.020* |
| Ipsi CA2 | -1.14 (1.61) | -1.03 (1.56) | <0.001** | <0.001** | <0.001** | 0.634 |
| Contra CA2 | -1.04 (2.43) | -0.79 (1.34) | <0.001** | <0.001** | <0.001** | 0.435 |
| Ipsi CA3 | -1.06 (1.30) | -0.61 (1.86) | <0.001** | <0.001** | <0.001** | 0.076 |
| Contra CA3 | -0.85 (1.61) | -0.42 (1.45) | <0.001** | <0.001** | 0.004* | 0.086 |
| Ipsi CA4 | -0.13 (1.54) | 0.05 (1.43) | 0.572 | 0.514 | 0.704 | 0.435 |
| Contra CA4 | -0.02 (1.31) | 0.28 (1.40) | 0.042* | 0.925 | 0.045* | 0.177 |
| Ipsi DG | -1.00 (1.59) | -0.39 (1.84) | <0.001** | <0.001** | 0.028* | 0.038* |
| Contra DG | -0.73 (1.58) | -0.06 (1.37) | <0.001** | <0.001** | 0.648 | 0.020* |
| Ipsi SRLM | -1.20 (1.52) | -0.65 (1.79) | <0.001** | <0.001** | <0.001** | 0.052 |
| Contra SRLM | -0.90 (1.50) | -0.31 (1.38) | <0.001** | <0.001** | 0.025* | 0.024* |

Comparison of hippocampal morphological Z-scores among healthy controls (N=424), patients with valproate exposure >6 months (N=70), and valproate-naive patients (N=134). Patients with short-term exposure (<6 months) were excluded. P-values were derived from one-way ANOVA (overall group differences) and post hoc Welch's t-tests (pairwise comparisons). All p-values were corrected for multiple comparisons using the Benjamini-Hochberg False Discovery Rate (FDR) method.

*P < 0.05, **P < 0.001.

Ipsi, ipsilateral; Contra, contralateral; CA, cornu ammonis; DG, dentate gyrus; SRLM, stratum radiatum/lacunosum-moleculare.

**Supplementary Table S5. Linear mixed-effects models of disease duration and valproate exposure predicting hippocampal morphological Z-score metrics in patients**

| **Variable** | β(Duration) | β(VPA) | P (Duration) | P (VPA) |
| --- | --- | --- | --- | --- |
| **Total Hippocampus Metrics** |  |  |  |  |
| Ipsi volume | -0.071 | -0.672 | 0.006* | 0.021* |
| Contra volume | -0.023 | -0.592 | 0.233 | 0.021* |
| Ipsi thickness | -0.051 | -0.42 | 0.011* | 0.069 |
| Contra thickness | -0.029 | -0.523 | 0.099 | 0.021* |
| Ipsi gyrification | -0.067 | -0.602 | 0.008* | 0.041* |
| Contra gyrification | -0.009 | -0.505 | 0.673 | 0.041* |
| Ipsi mean curvature | 0.037 | 0.357 | 0.183 | 0.249 |
| Contra mean curvature | 0.04 | 0.291 | 0.042* | 0.148 |
| Ipsi intrinsic curvature | 0.074 | 0.23 | 0.008* | 0.444 |
| Contra intrinsic curvature | 0.014 | 0.499 | 0.635 | 0.118 |
| **Subfield Volumes** |  |  |  |  |
| Ipsi subiculum | -0.045 | -0.518 | 0.015* | 0.021* |
| Contra subiculum | -0.023 | -0.3 | 0.183 | 0.148 |
| Ipsi CA1 | -0.076 | -0.534 | <0.001** | 0.034* |
| Contra CA1 | -0.025 | -0.517 | 0.183 | 0.021* |
| Ipsi CA2 | -0.058 | -0.116 | 0.008* | 0.612 |
| Contra CA2 | -0.009 | -0.129 | 0.673 | 0.612 |
| Ipsi CA3 | -0.048 | -0.493 | 0.042* | 0.069 |
| Contra CA3 | -0.028 | -0.326 | 0.183 | 0.165 |
| Ipsi CA4 | -0.04 | -0.301 | 0.052 | 0.188 |
| Contra CA4 | -0.008 | -0.235 | 0.673 | 0.286 |
| Ipsi DG | -0.084 | -0.756 | <0.001** | 0.021* |
| Contra DG | -0.017 | -0.585 | 0.421 | 0.021* |
| Ipsi SRLM | -0.066 | -0.653 | 0.007* | 0.021* |
| Contra SRLM | -0.025 | -0.452 | 0.183 | 0.046* |

Linear mixed-effects models were fitted separately for each hippocampal metric (Z-score) in the patient subset with complete medication records (n = 204). Fixed effects included disease duration (years) and valproate (VPA) exposure. Site was modeled as a random intercept. Reported β coefficients represent the estimated change in Z-score per 1-year increase in duration and the estimated mean Z-score difference between VPA-exposed and VPA-naïve patients. P-values were corrected for multiple comparisons across all metrics using the Benjamini–Hochberg false discovery rate (FDR) procedure, applied separately for duration and VPA effects.

Ipsi, ipsilateral; Contra, contralateral; CA, cornu ammonis; DG, dentate gyrus; SRLM, stratum radiatum/lacunosum-moleculare; VPA, valproate; FDR, false discovery rate.

**Supplementary Table S6. Paired Comparison of Ipsilateral and Contralateral Hippocampal Z-Score Metrics in Focal Epilepsy Patients**

| **Metric** | **Ipsi**  **Mean (SD)** | **Contra**  **Mean (SD)** | **t-statistic** | **P-value**  **(raw)** | **P-value**  **(FDR)** |
| --- | --- | --- | --- | --- | --- |
| **Total Hippocampus Metrics** |  |  |  |  |  |
| Volume | -1.35 (1.84) | -0.41 (1.37) | -15.65 | <0.001** | <0.001** |
| Thickness | -0.88 (1.73) | -0.28 (1.29) | -11.62 | <0.001** | <0.001** |
| Gyrification | -1.44 (2.11) | -0.27 (1.39) | -15.64 | <0.001** | <0.001** |
| Mean Curvature | 1.13 (2.30) | 0.19 (1.31) | 10.71 | <0.001** | <0.001** |
| Intrinsic Curvature | 1.55 (2.70) | 0.41 (1.85) | 11.47 | <0.001** | <0.001** |
| **Subfield Volumes** |  |  |  |  |  |
| Subiculum | -1.10 (1.50) | -0.35 (1.31) | -14.90 | <0.001** | <0.001** |
| CA1 | -1.48 (1.98) | -0.40 (1.38) | -14.49 | <0.001** | <0.001** |
| CA2 | -1.00 (2.03) | -0.54 (1.57) | -5.43 | <0.001** | <0.001** |
| CA3 | -1.18 (2.04) | -0.34 (1.54) | -10.06 | <0.001** | <0.001** |
| CA4 | -0.64 (1.78) | 0.15 (1.40) | -10.37 | <0.001** | <0.001** |
| DG | -1.49 (2.32) | -0.22 (1.46) | -15.16 | <0.001** | <0.001** |
| SRLM | -1.57 (2.23) | -0.31 (1.43) | -15.90 | <0.001** | <0.001** |

Paired comparison of ipsilateral and contralateral hippocampal Z-score metrics in patients with focal epilepsy (N=705, including those with MRI-diagnosed hippocampal sclerosis).
Values are presented as mean (standard deviation) for each side.
Paired t-tests were used to assess within-subject differences; p-values were corrected for multiple comparisons using the Benjamini-Hochberg False Discovery Rate (FDR) method.
*p < 0.05, **p < 0.001.
Ipsi, ipsilateral; Contra, contralateral; CA, cornu ammonis subfield; DG, dentate gyrus; SRLM, stratum radiatum/lacunosum-moleculare.

**Supplementary Table S7. Spearman Correlation Between Disease Duration and Hippocampal Z-Score Metrics in Patients Without MRI-Diagnosed Hippocampal Sclerosis**

| **Measure** | **Spearman ρ** | **p-value (raw)** | **p-value (FDR)** |
| --- | --- | --- | --- |
| **Total Hippocampus Metrics** |  |  |  |
| Ipsi volume | -0.130 | 0.004* | 0.042* |
| Contra volume | -0.120 | 0.007* | 0.042* |
| Ipsi thickness | -0.074 | 0.102 | 0.136 |
| Contra thickness | -0.100 | 0.026* | 0.069 |
| Ipsi gyrification | -0.069 | 0.124 | 0.156 |
| Contra gyrification | -0.042 | 0.353 | 0.386 |
| Ipsi mean curvature | 0.079 | 0.080 | 0.127 |
| Contra mean curvature | 0.077 | 0.088 | 0.132 |
| Ipsi intrinsic curvature | 0.123 | 0.006* | 0.042* |
| Contra intrinsic curvature | 0.124 | 0.006* | 0.042* |
| **Subfield Volumes** |  |  |  |
| Ipsi subiculum | -0.106 | 0.018* | 0.063 |
| Contra subiculum | -0.090 | 0.045* | 0.095 |
| Ipsi CA1 | -0.113 | 0.012* | 0.049* |
| Contra CA1 | -0.118 | 0.009* | 0.042* |
| Ipsi CA2 | -0.101 | 0.024* | 0.069 |
| Contra CA2 | -0.075 | 0.096 | 0.135 |
| Ipsi CA3 | -0.080 | 0.075 | 0.127 |
| Contra CA3 | -0.094 | 0.036* | 0.086 |
| Ipsi CA4 | 0.005 | 0.904 | 0.904 |
| Contra CA4 | -0.017 | 0.707 | 0.737 |
| Ipsi DG | -0.059 | 0.192 | 0.230 |
| Contra DG | -0.049 | 0.282 | 0.322 |
| Ipsi SRLM | -0.089 | 0.048* | 0.095 |
| Contra SRLM | -0.086 | 0.055 | 0.101 |

Spearman rank correlation coefficients (ρ) between disease duration and hippocampal Z-score metrics in patients without MRI-diagnosed hippocampal sclerosis (N=494).
P-values were adjusted for multiple comparisons using the Benjamini-Hochberg False Discovery Rate (FDR) method (α = 0.05).
*p < 0.05
Ipsi, ipsilateral; Contra, contralateral; CA, cornu ammonis subfield; DG, dentate gyrus; SRLM, stratum radiatum/lacunosum-moleculare.

**Supplementary Table S8. Independence between disease duration and age: original, age-partial, and age-stratified correlations**

| **Metric** | **Original Spearman ρ (P_FDR_)** | **Partial Spearman (Control Age) ρ (P_FDR_)** | **Pediatric (<18y) (N=257) ρ (P_FDR_)** | **Adult (≥18y) (N=448) ρ (P_FDR_)** |
| --- | --- | --- | --- | --- |
| **Total Hippocampus** |  |  |  |  |
| Ipsi volume | -0.255 (<0.001**) | -0.098 (0.044*) | -0.053 (0.838) | -0.275 (<0.001**) |
| Contra volume | 0.070 (0.077) | -0.034 (0.556) | 0.051 (0.838) | -0.084 (0.116) |
| Ipsi thickness | -0.332 (<0.001**) | -0.079 (0.089) | -0.023 (0.953) | -0.236 (<0.001**) |
| Contra thickness | -0.124 (0.002*) | 0.007 (0.935) | -0.030 (0.934) | -0.076 (0.154) |
| Ipsi gyrification | -0.276 (<0.001**) | -0.089 (0.064) | -0.028 (0.934) | -0.267 (<0.001**) |
| Contra gyrification | 0.070 (0.077) | -0.028 (0.650) | 0.089 (0.838) | -0.040 (0.451) |
| Ipsi mean curvature | 0.227 (<0.001**) | 0.024 (0.663) | 0.057 (0.838) | 0.158 (0.002*) |
| Contra mean curvature | -0.096 (0.014*) | 0.001 (0.973) | -0.016 (0.964) | 0.021 (0.658) |
| Ipsi intrinsic curvature | 0.231 (<0.001**) | 0.082 (0.077) | -0.009 (0.969) | 0.276 (<0.001**) |
| Contra intrinsic curvature | -0.103 (0.009*) | 0.009 (0.935) | -0.047 (0.838) | 0.068 (0.193) |
| **Subfield Volumes** |  |  |  |  |
| Ipsi subiculum | -0.333 (<0.001**) | -0.093 (0.053) | -0.115 (0.838) | -0.246 (<0.001**) |
| Contra subiculum | -0.107 (0.007*) | -0.068 (0.157) | 0.027 (0.934) | -0.130 (0.011*) |
| Ipsi CA1 | -0.283 (<0.001**) | -0.086 (0.067) | -0.056 (0.838) | -0.248 (<0.001**) |
| Contra CA1 | 0.107 (0.007*) | -0.026 (0.656) | 0.030 (0.934) | -0.038 (0.456) |
| Ipsi CA2 | -0.148 (<0.001**) | -0.124 (0.009*) | -0.003 (0.980) | -0.221 (<0.001**) |
| Contra CA2 | 0.150 (<0.001**) | 0.016 (0.800) | -0.068 (0.838) | 0.031 (0.538) |
| Ipsi CA3 | -0.275 (<0.001**) | -0.140 (0.005*) | -0.002 (0.980) | -0.279 (<0.001**) |
| Contra CA3 | 0.039 (0.324) | -0.060 (0.209) | 0.011 (0.969) | -0.072 (0.169) |
| Ipsi CA4 | -0.276 (<0.001**) | -0.035 (0.556) | -0.055 (0.838) | -0.217 (<0.001**) |
| Contra CA4 | -0.011 (0.798) | -0.004 (0.951) | 0.020 (0.953) | -0.042 (0.447) |
| Ipsi DG | -0.333 (<0.001**) | -0.122 (0.009*) | -0.051 (0.838) | -0.281 (<0.001**) |
| Contra DG | -0.003 (0.934) | -0.062 (0.196) | 0.050 (0.838) | -0.098 (0.066) |
| Ipsi SRLM | -0.320 (<0.001**) | -0.110 (0.021*) | -0.051 (0.838) | -0.276 (<0.001**) |
| Contra SRLM | 0.043 (0.295) | -0.048 (0.348) | 0.080 (0.838) | -0.090 (0.091) |

This table summarizes Spearman rank correlations between epilepsy duration and hippocampal Z-score metrics in the patient cohort, reported as: (i) original (unadjusted) Spearman ρ with FDR-corrected significance; (ii) partial Spearman ρ after controlling for age at scan; and (iii) stratified Spearman ρ within pediatric (<18 years) and adult (≥18 years) subgroups (sample sizes shown in the column headers). P values were adjusted for multiple comparisons using the Benjamini–Hochberg false discovery rate (FDR) procedure (α = 0.05).

*P < 0.05; **P < 0.001.

Ipsi, ipsilateral; Contra, contralateral; CA, cornu ammonis subfield; DG, dentate gyrus; SRLM, stratum radiatum/lacunosum-moleculare.

**Supplementary Table S9. Continuous age × duration interaction effects on hippocampal Z-score metrics (cluster-robust SE)**

| **Metric** | **β (Age×Dur)** | **SE** | **P (FDR)** |
| --- | --- | --- | --- |
| **Total Hippocampus Metrics** |  |  |  |
| Ipsi volume | 0.0009 | 0.0003 | 0.122 |
| Contra volume | 0.0011 | 0.0003 | 0.121 |
| Ipsi thickness | 0.0011 | 0.0004 | 0.123 |
| Contra thickness | 0.0013 | 0.0003 | 0.121 |
| Ipsi gyrification | 0.0001 | 0.0004 | 0.910 |
| Contra gyrification | 0.0004 | 0.0005 | 0.616 |
| Ipsi mean curvature | -0.0017 | 0.0008 | 0.192 |
| Contra mean curvature | -0.0008 | 0.0004 | 0.194 |
| Ipsi intrinsic curvature | -0.0001 | 0.0004 | 0.828 |
| Contra intrinsic curvature | -0.0006 | 0.0002 | 0.123 |
| **Subfield Volumes** |  |  |  |
| Ipsi subiculum | 0.0005 | 0.0002 | 0.189 |
| Contra subiculum | 0.0007 | 0.0004 | 0.220 |
| Ipsi CA1 | 0.0003 | 0.0002 | 0.377 |
| Contra CA1 | 0.0007 | 0.0002 | 0.121 |
| Ipsi CA2 | 0.0019 | 0.0006 | 0.122 |
| Contra CA2 | 0.0012 | 0.0004 | 0.131 |
| Ipsi CA3 | -0.0000 | 0.0004 | 0.910 |
| Contra CA3 | 0.0001 | 0.0003 | 0.828 |
| Ipsi CA4 | 0.0008 | 0.0002 | 0.121 |
| Contra CA4 | 0.0009 | 0.0003 | 0.123 |
| Ipsi DG | -0.0001 | 0.0002 | 0.632 |
| Contra DG | 0.0002 | 0.0004 | 0.699 |
| Ipsi SRLM | 0.0002 | 0.0002 | 0.632 |
| Contra SRLM | 0.0002 | 0.0003 | 0.632 |

This table evaluates whether the association between epilepsy duration and hippocampal Z-score metrics varies as a function of age by including a continuous interaction term (Age × Duration) in linear models. Age at scan and disease duration were mean-centered prior to computing the interaction term to reduce multicollinearity. Site and sex were included as categorical covariates (fixed effects). A non-significant p value indicates no robust evidence that age modifies the duration–metric association.

Ipsi, ipsilateral; Contra, contralateral; CA, cornu ammonis subfield; DG, dentate gyrus; SRLM, stratum radiatum/lacunosum-moleculare.

**Supplementary Table S10. Sensitivity analysis of disease duration–hippocampal correlations after adjustment for age at seizure onset**

| **Feature** | **ρ (unadjusted)** | **P_FDR_ (unadjusted)** | **ρ_partial**  **(onset-adjusted)** | **P_FDR_**  **(onset-adjusted)** |
| --- | --- | --- | --- | --- |
| **Total Hippocampus Metrics** |  |  |  |  |
| Ipsi volume | -0.255 | <0.001** | -0.253 | <0.001** |
| Contra volume | +0.070 | 0.077 | +0.080 | 0.042* |
| Ipsi thickness | -0.332 | <0.001** | -0.338 | <0.001** |
| Contra thickness | -0.124 | 0.002* | -0.132 | <0.001** |
| Ipsi gyrification | -0.276 | <0.001** | -0.277 | <0.001** |
| Contra gyrification | +0.070 | 0.077 | +0.079 | 0.045* |
| Ipsi mean curvature | +0.227 | <0.001** | +0.232 | <0.001** |
| Contra mean curvature | -0.096 | 0.014* | -0.102 | 0.009* |
| Ipsi intrinsic curvature | +0.231 | <0.001** | +0.230 | <0.001** |
| Contra intrinsic curvature | -0.103 | 0.009* | -0.114 | 0.004* |
| **Subfield Volumes** |  |  |  |  |
| Ipsi subiculum | -0.333 | <0.001** | -0.339 | <0.001** |
| Contra subiculum | -0.107 | 0.007* | -0.106 | 0.007* |
| Ipsi CA1 | -0.283 | <0.001** | -0.286 | <0.001** |
| Contra CA1 | +0.107 | 0.007* | +0.117 | 0.003* |
| Ipsi CA2 | -0.148 | <0.001** | -0.138 | <0.001** |
| Contra CA2 | +0.150 | <0.001** | +0.157 | <0.001** |
| Ipsi CA3 | -0.275 | <0.001** | -0.271 | <0.001** |
| Contra CA3 | +0.039 | 0.324 | +0.049 | 0.210 |
| Ipsi CA4 | -0.276 | <0.001** | -0.286 | <0.001** |
| Contra CA4 | -0.011 | 0.798 | -0.013 | 0.770 |
| Ipsi DG | -0.333 | <0.001** | -0.335 | <0.001** |
| Contra DG | -0.003 | 0.934 | +0.004 | 0.922 |
| Ipsi SRLM | -0.320 | <0.001** | -0.322 | <0.001** |
| Contra SRLM | +0.043 | 0.295 | +0.051 | 0.199 |

Partial Spearman correlations were computed by rank-transforming all three variables (disease duration, hippocampal metric, and age at seizure onset), residualizing the ranked duration and ranked metric on ranked onset age, and correlating the residuals. P-values for partial correlations were derived from the t-distribution with df = N − 3.

FDR, false discovery rate (Benjamini–Hochberg); Ipsi, ipsilateral; Contra, contralateral; CA, cornu ammonis; DG, dentate gyrus; SRLM, stratum radiatum/lacunosum-moleculare.

**Supplementary Table S11 Group Comparison of Hippocampal Z-Score Metrics by Disease Duration in Patients Without MRI-Diagnosed Hippocampal Sclerosis**

| **Variable** | **Shorter Duration Mean (SD)** | **Longer Duration Mean (SD)** | **P-value (ANOVA)** | **P Control vs Shorter** | **P Control vs Longer** | **P Shorter vs Longer** |
| --- | --- | --- | --- | --- | --- | --- |
| **Total Hippocampus Metrics** |  |  |  |  |  |  |
| Ipsi volume | -0.79 (1.76) | -0.64 (1.55) | <0.001** | <0.001** | <0.001** | 0.482 |
| Contra volume | -0.60 (1.56) | -0.38 (1.38) | <0.001** | <0.001** | <0.001** | 0.247 |
| Ipsi thickness | -0.21 (1.36) | -0.35 (1.34) | 0.001* | 0.039* | 0.001** | 0.482 |
| Contra thickness | -0.12 (1.26) | -0.24 (1.33) | 0.035* | 0.216 | 0.015* | 0.482 |
| Ipsi gyrification | -0.72 (1.85) | -0.49 (1.44) | <0.001** | <0.001** | <0.001** | 0.312 |
| Contra gyrification | -0.44 (1.59) | -0.25 (1.33) | <0.001** | <0.001** | 0.014* | 0.327 |
| Ipsi mean curvature | 0.45 (1.53) | 0.19 (1.71) | <0.001** | <0.001** | 0.120 | 0.225 |
| Contra mean curvature | 0.42 (1.30) | 0.06 (1.30) | <0.001** | <0.001** | 0.565 | 0.059 |
| Ipsi intrinsic curvature | 0.84 (1.99) | 0.67 (1.88) | <0.001** | <0.001** | <0.001** | 0.482 |
| Contra intrinsic curvature | 0.67 (1.88) | 0.35 (1.73) | <0.001** | <0.001** | 0.005* | 0.188 |
| **Subfield Volumes** |  |  |  |  |  |  |
| Ipsi subiculum | -0.46 (1.33) | -0.79 (1.41) | <0.001** | <0.001** | <0.001** | 0.063 |
| Contra subiculum | -0.19 (1.25) | -0.42 (1.37) | <0.001** | 0.047* | <0.001** | 0.188 |
| Ipsi CA1 | -0.76 (1.55) | -0.61 (1.57) | <0.001** | <0.001** | <0.001** | 0.482 |
| Contra CA1 | -0.62 (1.38) | -0.37 (1.44) | <0.001** | <0.001** | 0.001** | 0.188 |
| Ipsi CA2 | -0.82 (1.49) | -0.40 (2.22) | <0.001** | <0.001** | 0.012* | 0.081 |
| Contra CA2 | -0.82 (1.68) | -0.42 (1.57) | <0.001** | <0.001** | <0.001** | 0.063 |
| Ipsi CA3 | -0.50 (1.62) | -0.41 (1.74) | <0.001** | <0.001** | 0.002* | 0.667 |
| Contra CA3 | -0.41 (1.47) | -0.37 (1.62) | <0.001** | <0.001** | 0.003* | 0.809 |
| Ipsi CA4 | 0.01 (1.48) | 0.04 (1.55) | 0.909 | 0.962 | 0.689 | 0.809 |
| Contra CA4 | 0.08 (1.34) | 0.16 (1.43) | 0.264 | 0.425 | 0.131 | 0.667 |
| Ipsi DG | -0.45 (1.71) | -0.53 (1.60) | <0.001** | <0.001** | <0.001** | 0.763 |
| Contra DG | -0.27 (1.48) | -0.27 (1.50) | 0.007* | 0.014* | 0.014* | 0.999 |
| Ipsi SRLM | -0.64 (1.66) | -0.59 (1.70) | <0.001** | <0.001** | <0.001** | 0.809 |
| Contra SRLM | -0.43 (1.43) | -0.31 (1.54) | <0.001** | <0.001** | 0.007* | 0.501 |

This table presents group means and standard deviations (SD) for hippocampal global and subfield Z-score metrics across three groups: healthy controls (N=424), epilepsy patients with shorter disease duration (N=246), and epilepsy patients with longer disease duration (N=248). All patients with MRI-confirmed hippocampal sclerosis (HS) were excluded from this analysis. Group comparisons were performed using one-way ANOVA with post hoc Welch's t-tests. P-values were corrected for multiple comparisons using the Benjamini-Hochberg False Discovery Rate (FDR) method.

*P < 0.05; **P < 0.001.

Ipsi, ipsilateral; Contra, contralateral; CA, cornu ammonis subfield; DG, dentate gyrus; SRLM, stratum radiatum/lacunosum-moleculare.

**Supplementary Table S12. Baseline Characteristics of Longitudinal and Cross-sectional Patient Cohorts**

| **Characteristic** | **Longitudinal Cohort (N=80)** | **Cross-sectional Only Cohort (N=625)** | **P value** |
| --- | --- | --- | --- |
| Age (years), mean ± SD | 18.8 ± 15.2 | 26.2 ± 14.7 | <0.001* |
| Sex, female/male (n) | 49/31 | 330/295 | 0.191 |
| Age at onset (years), mean ± SD | 9.0 ± 8.7 | 10.7 ± 8.5 | 0.088 |
| Disease duration (years), mean ± SD | 9.9 ± 12.6 | 15.5 ± 13.6 | <0.001* |
| Lateralization, Left/Right (n) | 33/47 | 340/285 | 0.036* |
| Localization of seizure focus, n (%) |  |  | 0.754 |
| FL | 21 (26.2%) | 142 (22.7%) |  |
| IL | 0 (0.0%) | 3 (0.5%) |  |
| OL | 1 (1.2%) | 17 (2.7%) |  |
| PL | 6 (7.5%) | 43 (6.9%) |  |
| TL | 47 (58.8%) | 396 (63.4%) |  |
| U | 5 (6.2%) | 24 (3.8%) |  |

Comparison of baseline demographic and clinical characteristics between the longitudinal cohort (N=80, patients with two MRI scans) and the cross-sectional only cohort (N=625, patients with a single MRI scan). P-values were calculated using the two-sample t-test for continuous variables and the chi-square test for categorical variables.
*p < 0.05.
FL, frontal lobe; IL, insular lobe; L, left; OL, occipital lobe; PL, parietal lobe; R, right; SD, standard deviation; TL, temporal lobe; U, undetermined.

**Supplementary Table S13. Distribution of individual annualized hippocampal Z-score changes stratified by drug-resistant epilepsy status**

| **Feature** | **Total (N=80)** | **Non-DRE (N=27)** | **DRE (N=53)** | **P-value (FDR)** |
| --- | --- | --- | --- | --- |
| **Total Hippocampus Metrics** |  |  |  |  |
| Ipsi Volume | -0.24 (-0.59, -0.01) | -0.37 (-0.68, -0.06) | -0.24 (-0.45, -0.01) | 0.848 |
| Contra Volume | -0.24 (-0.61, 0.03) | -0.24 (-0.66, -0.07) | -0.21 (-0.61, 0.10) | 0.848 |
| Ipsi Thickness | 0.14 (-0.31, 0.51) | -0.18 (-0.45, 0.32) | 0.28 (-0.20, 0.77) | 0.101 |
| Contra Thickness | 0.17 (-0.22, 0.67) | 0.20 (-0.21, 0.62) | 0.14 (-0.23, 0.71) | 0.848 |
| Ipsi Gyrification | -0.01 (-0.36, 0.32) | -0.19 (-0.54, 0.23) | 0.09 (-0.19, 0.48) | 0.101 |
| Contra Gyrification | -0.14 (-0.51, 0.22) | -0.28 (-0.56, 0.13) | -0.12 (-0.49, 0.22) | 0.848 |
| Ipsi Mean Curvature | -0.14 (-0.76, 0.47) | 0.25 (-0.27, 0.62) | -0.21 (-0.84, 0.45) | 0.529 |
| Contra Mean Curvature | -0.20 (-0.59, 0.22) | 0.16 (-0.32, 0.40) | -0.26 (-0.76, 0.19) | 0.106 |
| Ipsi Intrinsic Curvature | 0.51 (-0.21, 0.82) | 0.52 (0.09, 0.64) | 0.51 (-0.33, 0.89) | 0.976 |
| Contra Intrinsic Curvature | -0.05 (-0.54, 0.95) | -0.12 (-0.54, 0.46) | 0.00 (-0.53, 1.08) | 0.848 |
| **Subfield Volumes** |  |  |  |  |
| Ipsi Subiculum | -0.22 (-0.62, 0.20) | -0.06 (-0.46, 0.36) | -0.25 (-0.80, 0.16) | 0.184 |
| Contra Subiculum | -0.24 (-0.88, 0.19) | -0.20 (-0.54, 0.21) | -0.24 (-0.91, 0.10) | 0.848 |
| Ipsi CA1 | -0.20 (-0.60, 0.11) | -0.36 (-0.65, -0.08) | -0.17 (-0.53, 0.15) | 0.666 |
| Contra CA1 | -0.06 (-0.22, 0.45) | -0.13 (-0.19, 0.30) | -0.02 (-0.24, 0.53) | 0.848 |
| Ipsi CA2 | -0.28 (-1.26, 0.51) | -0.62 (-1.41, 0.20) | 0.29 (-0.72, 0.55) | 0.101 |
| Contra CA2 | 0.10 (-0.73, 0.55) | -0.26 (-0.69, 0.51) | 0.13 (-0.82, 0.61) | 0.848 |
| Ipsi CA3 | -0.23 (-1.04, 0.24) | -0.23 (-0.95, 0.25) | -0.23 (-1.08, 0.24) | 0.848 |
| Contra CA3 | -0.11 (-0.69, 0.16) | -0.09 (-0.43, 0.13) | -0.11 (-1.17, 0.18) | 0.848 |
| Ipsi CA4 | -0.07 (-0.51, 0.55) | -0.05 (-0.61, 0.43) | -0.09 (-0.39, 0.71) | 0.932 |
| Contra CA4 | -0.07 (-0.43, 0.44) | -0.36 (-0.51, -0.08) | 0.32 (-0.33, 0.51) | 0.031* |
| Ipsi DG | -0.32 (-0.51, 0.28) | -0.31 (-0.49, 0.22) | -0.33 (-0.50, 0.42) | 0.976 |
| Contra DG | -0.25 (-0.66, 0.27) | -0.24 (-0.74, 0.21) | -0.28 (-0.66, 0.28) | 0.931 |
| Ipsi SRLM | -0.19 (-0.55, 0.06) | -0.08 (-0.54, 0.10) | -0.23 (-0.56, 0.04) | 0.848 |
| Contra SRLM | -0.16 (-0.33, 0.23) | -0.09 (-0.33, 0.17) | -0.18 (-0.32, 0.23) | 0.848 |

Values are presented as median [interquartile range (IQR)] of individual annualized change (Z-score/year). Negative values indicate progressive deviation below age-matched healthy norms. Patients were stratified by drug-resistant epilepsy (DRE) status. Between-group comparisons were performed using the Wilcoxon rank-sum test (or t-test, as appropriate), and p-values were adjusted for multiple comparisons using the Benjamini–Hochberg false discovery rate (FDR) method.

DRE, drug-resistant epilepsy; Ipsi, ipsilateral; Contra, contralateral; CA, cornu ammonis; DG, dentate gyrus; SRLM, stratum radiatum/lacunosum-moleculare.

**Supplementary Table S14. Longitudinal Comparison of Hippocampal Z-Score Metrics Between Two MRI Scans in Focal Epilepsy Patients**

| **Variable** | **Scan 1**  **Mean (SD)** | **Scan 2**  **Mean (SD)** | **Percent Decreased (%)** | **t-statistic** | **P-value (FDR)** |
| --- | --- | --- | --- | --- | --- |
| **Total Hippocampus Metrics** |  |  |  |  |  |
| Ipsi Volume | -0.88 (1.53) | -1.20 (1.82) | 76.2% | -2.98 | 0.018* |
| Contra Volume | -0.52 (1.18) | -0.79 (1.49) | 72.5% | -2.82 | 0.024* |
| Ipsi Thickness | -0.47 (1.29) | -0.48 (1.60) | 43.8% | -0.11 | 0.956 |
| Contra Thickness | -0.32 (0.99) | -0.14 (1.14) | 32.5% | 2.21 | 0.079 |
| Ipsi Gyrification | -0.97 (1.87) | -1.13 (1.92) | 51.2% | -2.32 | 0.069 |
| Contra Gyrification | -0.43 (1.30) | -0.56 (1.48) | 66.2% | -1.99 | 0.109 |
| Ipsi Mean Curvature | 0.59 (1.67) | 0.48 (1.69) | 58.8% | -0.99 | 0.410 |
| Contra Mean Curvature | 0.24 (1.06) | 0.15 (1.18) | 55.0% | -1.03 | 0.410 |
| Ipsi Intrinsic Curvature | 1.12 (2.51) | 1.39 (2.93) | 28.7% | 1.65 | 0.176 |
| Contra Intrinsic Curvature | 0.54 (1.73) | 0.50 (2.04) | 52.5% | -0.28 | 0.850 |
| **Subfield Volumes** |  |  |  |  |  |
| Ipsi Subiculum | -0.75 (1.32) | -0.86 (1.49) | 61.3% | -1.28 | 0.329 |
| Contra Subiculum | -0.32 (1.07) | -0.70 (1.30) | 67.5% | -3.97 | 0.004* |
| Ipsi CA1 | -0.83 (1.54) | -1.13 (1.79) | 68.8% | -3.03 | 0.018* |
| Contra CA1 | -0.52 (1.11) | -0.62 (1.48) | 53.8% | -1.07 | 0.405 |
| Ipsi CA2 | -0.76 (1.47) | -1.06 (1.88) | 53.8% | -1.90 | 0.115 |
| Contra CA2 | -0.77 (1.29) | -0.86 (1.54) | 46.2% | -0.70 | 0.555 |
| Ipsi CA3 | -0.72 (1.58) | -0.81 (1.74) | 62.5% | -0.76 | 0.543 |
| Contra CA3 | -0.25 (1.18) | -0.39 (1.50) | 58.8% | -1.19 | 0.359 |
| Ipsi CA4 | -0.14 (1.49) | -0.38 (1.61) | 52.5% | -1.89 | 0.115 |
| Contra CA4 | 0.03 (1.30) | 0.02 (1.56) | 53.8% | -0.05 | 0.962 |
| Ipsi Dg | -0.79 (1.83) | -1.07 (2.01) | 65.0% | -3.08 | 0.018* |
| Contra Dg | -0.13 (1.16) | -0.43 (1.38) | 65.0% | -3.43 | 0.011* |
| Ipsi Srlm | -0.90 (1.76) | -1.11 (1.88) | 66.2% | -2.73 | 0.027* |
| Contra Srlm | -0.37 (1.14) | -0.55 (1.40) | 63.7% | -2.09 | 0.095 |

Paired comparison of hippocampal Z-score metrics between Scan 1 and Scan 2 in focal epilepsy patients with longitudinal data (N=80). Percent Decreased (%) indicates the percentage of subjects whose Z-score decreased from Scan 1 to Scan 2. Paired t-tests were used to assess within-subject changes; p-values were corrected for multiple comparisons using the Benjamini-Hochberg False Discovery Rate (FDR) method.
*p < 0.05.
Ipsi, ipsilateral; Contra, contralateral; CA, cornu ammonis subfield; DG, dentate gyrus; SRLM, stratum radiatum/lacunosum-moleculare.

**Supplementary Table S15. Demographic and Clinical Characteristics of Focal Epilepsy Patients Without Non-HS Structural Lesions and Healthy Controls**

| **Characteristic** | **Focal epilepsy without Non-HS Lesions**  **(n=503)** | **Healthy controls**  **(n=424)** | **P value** |
| --- | --- | --- | --- |
| Sex, female/male | 278/225 | 235/189 | 1.000 |
| Age, mean ± SD | 24.6 ± 15.0 | 22.9 ± 16.5 | 0.089 |
| Onset of epilepsy, mean ± SD, years | 9.7 ± 8.0 | NA | NA |
| disease duration, mean ± SD, years | 15.0 ± 13.9 | NA | NA |
| Lateralization of seizure focus (n) |  |  | NA |
| L | 266 | NA |  |
| R | 237 | NA |  |
| Localization of seizure focus, or epilepsy type (n, %) |  | NA | NA |
| FL | 57 (30.6%) | NA |  |
| IL | 0 (0.0%) | NA |  |
| PL | 15 (8.1%) | NA |  |
| OL | 9 (4.8%) | NA |  |
| TL | 82 (44.1%) | NA |  |
| U | 23 (12.4%) | NA |  |

This table summarizes the demographic and clinical characteristics of focal epilepsy patients after exclusion of cases with structural lesions other than hippocampal sclerosis (HS), compared to healthy controls. Data are presented as mean ± standard deviation (SD) or number (percentage) as appropriate. "Onset of epilepsy" refers to the age at first seizure. " disease duration " refers to the interval between age at seizure onset and age at enrollment.

L, left; R, right; TL, temporal lobe; FL, frontal lobe; PL, parietal lobe; OL, occipital lobe; IL, insular lobe; U, undetermined lobe; NA, not applicable.

**Supplementary Table S16. Group Differences in Hippocampal Z-Score Metrics Between Focal Epilepsy Patients Without Non-HS Structural Lesions and Healthy Controls**

| **Metric** | **Mean (SD)** | **t-statistic** | **Cohen’s d** | **P-value (raw)** | **P-value (FDR)** |
| --- | --- | --- | --- | --- | --- |
| **Total Hippocampus Metrics** |  |  |  |  |  |
| Ipsi volume | -1.48 (1.85) | -15.47 | -1.02 | <0.001** | <0.001** |
| Contra volume | -0.41 (1.38) | -5.28 | -0.35 | <0.001** | <0.001** |
| Ipsi thickness | -0.98 (1.80) | -10.40 | -0.69 | <0.001** | <0.001** |
| Contra thickness | -0.30 (1.31) | -3.97 | -0.26 | <0.001** | <0.001** |
| Ipsi gyrification | -1.58 (2.12) | -14.86 | -0.98 | <0.001** | <0.001** |
| Contra gyrification | -0.25 (1.35) | -3.26 | -0.21 | 0.001* | 0.001* |
| Ipsi mean curvature | 1.28 (2.39) | 10.96 | 0.72 | <0.001** | <0.001** |
| Contra mean curvature | 0.23 (1.34) | 2.96 | 0.20 | 0.003* | 0.003* |
| Ipsi intrinsic curvature | 1.65 (2.63) | 12.97 | 0.86 | <0.001** | <0.001** |
| Contra intrinsic curvature | 0.39 (1.87) | 4.04 | 0.27 | <0.001** | <0.001** |
| **Subfield Volumes** |  |  |  |  |  |
| Ipsi subiculum | -1.15 (1.48) | -14.02 | -0.92 | <0.001** | <0.001** |
| Contra subiculum | -0.35 (1.27) | -4.66 | -0.31 | <0.001** | <0.001** |
| Ipsi CA1 | -1.62 (1.99) | -15.98 | -1.05 | <0.001** | <0.001** |
| Contra CA1 | -0.39 (1.40) | -4.90 | -0.32 | <0.001** | <0.001** |
| Ipsi CA2 | -1.12 (1.95) | -11.19 | -0.74 | <0.001** | <0.001** |
| Contra CA2 | -0.55 (1.43) | -6.90 | -0.45 | <0.001** | <0.001** |
| Ipsi CA3 | -1.31 (2.08) | -12.53 | -0.83 | <0.001** | <0.001** |
| Contra CA3 | -0.33 (1.55) | -3.85 | -0.25 | <0.001** | <0.001** |
| Ipsi CA4 | -0.80 (1.77) | -8.63 | -0.57 | <0.001** | <0.001** |
| Contra CA4 | 0.15 (1.40) | 1.86 | 0.12 | 0.063 | 0.063 |
| Ipsi DG | -1.66 (2.36) | -14.34 | -0.95 | <0.001** | <0.001** |
| Contra DG | -0.19 (1.45) | -2.39 | -0.16 | 0.017* | 0.018* |
| Ipsi SRLM | -1.75 (2.24) | -15.69 | -1.03 | <0.001** | <0.001** |
| Contra SRLM | -0.29 (1.41) | -3.66 | -0.24 | <0.001** | <0.001** |

Comparison of hippocampal Z-score metrics between focal epilepsy patients without non-hippocampal structural lesions (N=503) and healthy controls (N=424). This sensitivity analysis excluded patients with structural abnormalities other than hippocampal sclerosis to assess epilepsy-related hippocampal changes independent of confounding structural pathology.

Data are presented as mean ± standard deviation for the patient group. Group comparisons were performed using Welch's independent samples t-test. P-values were corrected for multiple comparisons using the Benjamini-Hochberg False Discovery Rate (FDR) method. Cohen’s d = t × √(1/n₁ + 1/n₂).

*P < 0.05, **P < 0.001
Ipsi, ipsilateral; Contra, contralateral; CA, cornu ammonis subfield; DG, dentate gyrus; SRLM, stratum radiatum/lacunosum-moleculare.

**Supplementary Table S17. Group Differences in Hippocampal Z-Score Metrics Between MRI-Negative Focal Epilepsy Patients and Healthy Controls**

| **Metric** | **Mean (SD)** | **t-statistic** | **Cohen’s d** | **P-value (raw)** | **P-value (FDR)** |
| --- | --- | --- | --- | --- | --- |
| **Total Hippocampus Metrics** |  |  |  |  |  |
| Ipsi volume | -0.67 (1.67) | -6.32 | -0.47 | <0.001** | <0.001** |
| Contra volume | -0.53 (1.54) | -5.35 | -0.40 | <0.001** | <0.001** |
| Ipsi thickness | -0.21 (1.35) | -2.30 | -0.17 | 0.022* | 0.025* |
| Contra thickness | -0.16 (1.31) | -1.77 | -0.13 | 0.078 | 0.085 |
| Ipsi gyrification | -0.51 (1.58) | -5.00 | -0.37 | <0.001** | <0.001** |
| Contra gyrification | -0.35 (1.43) | -3.66 | -0.27 | <0.001** | <0.001** |
| Ipsi mean curvature | 0.24 (1.52) | 2.43 | 0.18 | 0.015* | 0.018* |
| Contra mean curvature | 0.28 (1.33) | 3.13 | 0.23 | 0.002* | 0.003* |
| Ipsi intrinsic curvature | 0.65 (1.72) | 5.98 | 0.45 | <0.001** | <0.001** |
| Contra intrinsic curvature | 0.53 (1.79) | 4.71 | 0.35 | <0.001** | <0.001** |
| **Subfield Volumes** |  |  |  |  |  |
| Ipsi subiculum | -0.53 (1.31) | -5.96 | -0.44 | <0.001** | <0.001** |
| Contra subiculum | -0.27 (1.26) | -3.11 | -0.23 | 0.002* | 0.003* |
| Ipsi CA1 | -0.61 (1.50) | -6.25 | -0.47 | <0.001** | <0.001** |
| Contra CA1 | -0.51 (1.44) | -5.36 | -0.40 | <0.001** | <0.001** |
| Ipsi CA2 | -0.61 (1.83) | -5.32 | -0.40 | <0.001** | <0.001** |
| Contra CA2 | -0.69 (1.47) | -7.14 | -0.53 | <0.001** | <0.001** |
| Ipsi CA3 | -0.39 (1.65) | -3.67 | -0.27 | <0.001** | <0.001** |
| Contra CA3 | -0.40 (1.56) | -3.97 | -0.30 | <0.001** | <0.001** |
| Ipsi CA4 | 0.04 (1.43) | 0.47 | 0.04 | 0.636 | 0.636 |
| Contra CA4 | 0.11 (1.38) | 1.20 | 0.09 | 0.230 | 0.240 |
| Ipsi DG | -0.39 (1.56) | -3.91 | -0.29 | <0.001** | <0.001** |
| Contra DG | -0.27 (1.48) | -2.75 | -0.20 | 0.006* | 0.008* |
| Ipsi SRLM | -0.55 (1.60) | -5.33 | -0.40 | <0.001** | <0.001** |
| Contra SRLM | -0.37 (1.47) | -3.86 | -0.29 | <0.001** | <0.001** |

Comparison of hippocampal Z-score metrics between MRI-negative focal epilepsy patients (N=313) and healthy controls (N=424). The MRI-negative cohort was defined by excluding all patients with hippocampal sclerosis or any other structural lesions, representing patients with normal conventional MRI findings. Group comparisons were performed using Welch's independent samples t-test. P-values were corrected for multiple comparisons using the Benjamini-Hochberg False Discovery Rate (FDR) method. Cohen’s d = t × √(1/n₁ + 1/n₂).

*P < 0.05, **P < 0.001

Ipsi, ipsilateral; Contra, contralateral; CA, cornu ammonis subfield; DG, dentate gyrus; SRLM, stratum radiatum/lacunosum-moleculare.

**Supplementary Table S18.** **Paired Comparison of Ipsilateral and Contralateral Hippocampal Z-Score Metrics in Focal Epilepsy Patients Without Non-HS Structural Lesions**

| **Metric** | **Ipsi Mean (SD)** | **Contra Mean (SD)** | **t-statistic** | **P-value (raw)** | **P-value (FDR)** |
| --- | --- | --- | --- | --- | --- |
| **Total Hippocampus Metrics** |  |  |  |  |  |
| Volume | -1.48 (1.85) | -0.41 (1.38) | -14.76 | <0.001** | <0.001** |
| Thickness | -0.98 (1.80) | -0.30 (1.31) | -10.85 | <0.001** | <0.001** |
| Gyrification | -1.58 (2.12) | -0.25 (1.35) | -14.85 | <0.001** | <0.001** |
| Mean Curvature | 1.28 (2.39) | 0.23 (1.34) | 9.96 | <0.001** | <0.001** |
| Intrinsic Curvature | 1.65 (2.63) | 0.39 (1.87) | 10.71 | <0.001** | <0.001** |
| **Subfield Volumes** |  |  |  |  |  |
| Subiculum | -1.15 (1.48) | -0.35 (1.27) | -13.87 | <0.001** | <0.001** |
| CA1 | -1.62 (1.99) | -0.39 (1.40) | -13.87 | <0.001** | <0.001** |
| CA2 | -1.12 (1.95) | -0.55 (1.43) | -5.96 | <0.001** | <0.001** |
| CA3 | -1.31 (2.08) | -0.33 (1.55) | -9.79 | <0.001** | <0.001** |
| CA4 | -0.80 (1.77) | 0.15 (1.40) | -10.53 | <0.001** | <0.001** |
| DG | -1.66 (2.36) | -0.19 (1.45) | -14.55 | <0.001** | <0.001** |
| SRLM | -1.75 (2.24) | -0.29 (1.41) | -15.51 | <0.001** | <0.001** |

This table presents paired comparisons of global and subfield hippocampal Z-score metrics between the ipsilateral and contralateral hippocampus in focal epilepsy patients (N=503) after excluding those with structural lesions other than hippocampal sclerosis (HS). Paired-samples t-tests were used to assess within-subject differences. P-values were corrected for multiple comparisons using the Benjamini-Hochberg False Discovery Rate (FDR) method.
*p < 0.05; **p < 0.001.
Ipsi, ipsilateral; Contra, contralateral; CA, cornu ammonis subfield; DG, dentate gyrus; SRLM, stratum radiatum/lacunosum-moleculare.

**Supplementary Table S19. Paired Comparison of Ipsilateral and Contralateral Hippocampal Z-Score Metrics in MRI-Negative Focal Epilepsy Patients**

| **Metric** | **Ipsi Mean (SD)** | **Contra Mean (SD)** | **t-statistic** | **P-value (raw)** | **P-value (FDR)** |
| --- | --- | --- | --- | --- | --- |
| **Total Hippocampus Metrics** |  |  |  |  |  |
| Volume | -0.67 (1.67) | -0.53 (1.54) | -2.36 | 0.019* | 0.057 |
| Thickness | -0.21 (1.35) | -0.16 (1.31) | -1.06 | 0.289 | 0.434 |
| Gyrification | -0.51 (1.58) | -0.35 (1.43) | -2.51 | 0.012* | 0.050* |
| Mean Curvature | 0.24 (1.52) | 0.28 (1.33) | -0.56 | 0.573 | 0.625 |
| Intrinsic Curvature | 0.65 (1.72) | 0.53 (1.79) | 1.34 | 0.181 | 0.310 |
| **Subfield Volumes** |  |  |  |  |  |
| Subiculum | -0.53 (1.31) | -0.27 (1.26) | -4.49 | <0.001** | <0.001** |
| CA1 | -0.61 (1.50) | -0.51 (1.44) | -1.47 | 0.143 | 0.287 |
| CA2 | -0.61 (1.83) | -0.69 (1.47) | 0.79 | 0.432 | 0.569 |
| CA3 | -0.39 (1.65) | -0.40 (1.56) | 0.13 | 0.898 | 0.898 |
| CA4 | 0.04 (1.43) | 0.11 (1.38) | -0.72 | 0.474 | 0.569 |
| DG | -0.39 (1.56) | -0.27 (1.48) | -1.92 | 0.056 | 0.134 |
| SRLM | -0.55 (1.60) | -0.37 (1.47) | -2.95 | 0.003* | 0.020* |

This table presents paired comparisons of global and subfield hippocampal Z-score metrics between the ipsilateral (seizure focus side) and contralateral hippocampus in MRI-negative focal epilepsy patients (N=313). This cohort excludes all patients with hippocampal sclerosis or any other structural lesions. Paired-samples t-tests were used to assess within-subject differences. P-values were corrected for multiple comparisons using the Benjamini-Hochberg False Discovery Rate (FDR) method.

*P < 0.05, **P < 0.001

Ipsi, ipsilateral; Contra, contralateral; CA, cornu ammonis subfield; DG, dentate gyrus; SRLM, stratum radiatum/lacunosum-moleculare.

**Supplementary Table S20. Spearman Correlation Between Disease Duration and Hippocampal Z-Score Metrics in Focal Epilepsy Patients Without Non-HS Structural Lesions**

| **Measure** | **Spearman ρ** | **p-value (raw)** | **p-value (FDR)** |
| --- | --- | --- | --- |
| **Total Hippocampus Metrics** |  |  |  |
| Ipsi volume | -0.429 | <0.001** | <0.001** |
| Contra volume | 0.049 | 0.273 | 0.328 |
| Ipsi thickness | -0.440 | <0.001** | <0.001** |
| Contra thickness | -0.169 | <0.001** | <0.001** |
| Ipsi gyrification | -0.443 | <0.001** | <0.001** |
| Contra gyrification | 0.071 | 0.111 | 0.140 |
| Ipsi mean curvature | 0.361 | <0.001** | <0.001** |
| Contra mean curvature | -0.091 | 0.041* | 0.055 |
| Ipsi intrinsic curvature | 0.337 | <0.001** | <0.001** |
| Contra intrinsic curvature | -0.129 | 0.004* | 0.006* |
| **Subfield Volumes** |  |  |  |
| Ipsi subiculum | -0.448 | <0.001** | <0.001** |
| Contra subiculum | -0.153 | <0.001** | <0.001** |
| Ipsi CA1 | -0.441 | <0.001** | <0.001** |
| Contra CA1 | 0.099 | 0.026* | 0.037* |
| Ipsi CA2 | -0.237 | <0.001** | <0.001** |
| Contra CA2 | 0.164 | <0.001** | <0.001** |
| Ipsi CA3 | -0.411 | <0.001** | <0.001** |
| Contra CA3 | 0.018 | 0.680 | 0.680 |
| Ipsi CA4 | -0.468 | <0.001** | <0.001** |
| Contra CA4 | -0.043 | 0.340 | 0.372 |
| Ipsi DG | -0.498 | <0.001** | <0.001** |
| Contra DG | -0.043 | 0.341 | 0.372 |
| Ipsi SRLM | -0.491 | <0.001** | <0.001** |
| Contra SRLM | 0.028 | 0.537 | 0.561 |

Spearman rank correlation analysis examining associations between disease duration and hippocampal Z-score metrics in focal epilepsy patients without non-hippocampal structural lesions (N=502). This cohort includes patients with hippocampal sclerosis but excludes those with other structural abnormalities. P-values were corrected for multiple comparisons using the Benjamini-Hochberg False Discovery Rate (FDR) method.

*P < 0.05, **P < 0.001

Ipsi, ipsilateral; Contra, contralateral; CA, cornu ammonis subfield; DG, dentate gyrus; SRLM, stratum radiatum/lacunosum-moleculare.

**Supplementary Table S21. Spearman Correlation Between Disease Duration and Hippocampal Z-Score Metrics in MRI-Negative Focal Epilepsy Patients**

| **Measure** | **Spearman ρ** | **p-value (raw)** | **p-value (FDR)** |
| --- | --- | --- | --- |
| **Total Hippocampus Metrics** |  |  |  |
| Ipsi volume | -0.138 | 0.015* | 0.051 |
| Contra volume | -0.000 | 0.995 | 0.995 |
| Ipsi thickness | -0.194 | <0.001** | 0.007* |
| Contra thickness | -0.153 | 0.007* | 0.027* |
| Ipsi gyrification | -0.104 | 0.066 | 0.122 |
| Contra gyrification | 0.023 | 0.691 | 0.789 |
| Ipsi mean curvature | 0.008 | 0.894 | 0.933 |
| Contra mean curvature | -0.116 | 0.040* | 0.106 |
| Ipsi intrinsic curvature | 0.043 | 0.446 | 0.535 |
| Contra intrinsic curvature | -0.102 | 0.071 | 0.122 |
| **Subfield Volumes** |  |  |  |
| Ipsi subiculum | -0.241 | <0.001** | <0.001** |
| Contra subiculum | -0.169 | 0.003* | 0.013* |
| Ipsi CA1 | -0.104 | 0.068 | 0.122 |
| Contra CA1 | 0.062 | 0.276 | 0.349 |
| Ipsi CA2 | 0.062 | 0.274 | 0.349 |
| Contra CA2 | 0.104 | 0.067 | 0.122 |
| Ipsi CA3 | -0.088 | 0.122 | 0.194 |
| Contra CA3 | -0.073 | 0.200 | 0.299 |
| Ipsi CA4 | -0.129 | 0.023* | 0.069 |
| Contra CA4 | -0.065 | 0.249 | 0.349 |
| Ipsi DG | -0.180 | 0.001* | 0.010* |
| Contra DG | -0.114 | 0.044* | 0.106 |
| Ipsi SRLM | -0.178 | 0.002* | 0.010* |
| Contra SRLM | -0.017 | 0.763 | 0.832 |

Spearman rank correlation analysis examining associations between disease duration and hippocampal Z-score metrics in MRI-negative focal epilepsy patients (N=312). This cohort excludes all patients with hippocampal sclerosis or any other structural lesions. P-values were corrected for multiple comparisons using the Benjamini-Hochberg False Discovery Rate (FDR) method.

*P < 0.05, **P < 0.001

Ipsi, ipsilateral; Contra, contralateral; CA, cornu ammonis subfield; DG, dentate gyrus; SRLM, stratum radiatum/lacunosum-moleculare.

**Supplementary Table S22. Disease Duration Subgroup Comparison in Focal Epilepsy Patients Without Non-HS Structural Lesions**

| **Metric** | **Shorter Duration Mean (SD)** | **Longer Duration Mean (SD)** | **P-value (ANOVA)** | **P Control vs Shorter** | **P Control vs Longer** | **P Shorter vs Longer** |
| --- | --- | --- | --- | --- | --- | --- |
| **Total Hippocampus Metrics** |  |  |  |  |  |  |
| Ipsi volume | -0.88 (1.81) | -2.08 (1.68) | <0.001** | <0.001** | <0.001** | <0.001** |
| Contra volume | -0.50 (1.61) | -0.33 (1.11) | <0.001** | <0.001** | <0.001** | 0.193 |
| Ipsi thickness | -0.31 (1.55) | -1.64 (1.80) | <0.001** | 0.006* | <0.001** | <0.001** |
| Contra thickness | -0.13 (1.39) | -0.48 (1.21) | <0.001** | 0.215 | <0.001** | 0.005* |
| Ipsi gyrification | -0.82 (1.86) | -2.34 (2.09) | <0.001** | <0.001** | <0.001** | <0.001** |
| Contra gyrification | -0.34 (1.50) | -0.16 (1.17) | 0.002* | 0.002* | 0.077 | 0.183 |
| Ipsi mean curvature | 0.54 (1.73) | 2.03 (2.70) | <0.001** | <0.001** | <0.001** | <0.001** |
| Contra mean curvature | 0.31 (1.34) | 0.14 (1.32) | 0.005* | 0.002* | 0.150 | 0.190 |
| Ipsi intrinsic curvature | 0.94 (2.01) | 2.35 (2.97) | <0.001** | <0.001** | <0.001** | <0.001** |
| Contra intrinsic curvature | 0.59 (1.95) | 0.19 (1.76) | <0.001** | <0.001** | 0.138 | 0.022* |
| **Subfield Volumes** |  |  |  |  |  |  |
| Ipsi subiculum | -0.63 (1.44) | -1.67 (1.35) | <0.001** | <0.001** | <0.001** | <0.001** |
| Contra subiculum | -0.19 (1.26) | -0.51 (1.26) | <0.001** | 0.054 | <0.001** | 0.006* |
| Ipsi CA1 | -0.91 (1.74) | -2.32 (1.97) | <0.001** | <0.001** | <0.001** | <0.001** |
| Contra CA1 | -0.50 (1.47) | -0.27 (1.31) | <0.001** | <0.001** | 0.007* | 0.085 |
| Ipsi CA2 | -0.75 (1.90) | -1.48 (1.94) | <0.001** | <0.001** | <0.001** | <0.001** |
| Contra CA2 | -0.76 (1.49) | -0.34 (1.34) | <0.001** | <0.001** | 0.001** | 0.002* |
| Ipsi CA3 | -0.61 (1.72) | -2.01 (2.17) | <0.001** | <0.001** | <0.001** | <0.001** |
| Contra CA3 | -0.38 (1.53) | -0.27 (1.57) | 0.001** | 0.001** | 0.019* | 0.469 |
| Ipsi CA4 | -0.10 (1.56) | -1.50 (1.68) | <0.001** | 0.381 | <0.001** | <0.001** |
| Contra CA4 | 0.21 (1.41) | 0.08 (1.39) | 0.092 | 0.042* | 0.424 | 0.324 |
| Ipsi DG | -0.69 (1.88) | -2.64 (2.39) | <0.001** | <0.001** | <0.001** | <0.001** |
| Contra DG | -0.16 (1.51) | -0.23 (1.39) | 0.060 | 0.153 | 0.028* | 0.589 |
| Ipsi SRLM | -0.86 (1.91) | -2.64 (2.20) | <0.001** | <0.001** | <0.001** | <0.001** |
| Contra SRLM | -0.33 (1.50) | -0.25 (1.31) | 0.002* | 0.002* | 0.014* | 0.506 |

Comparison of hippocampal Z-score metrics among healthy controls (N=424), shorter disease duration (N=251), and longer disease duration (N=251) groups after excluding patients with non-hippocampal structural lesions. Patients were divided using the same disease duration median split as in the main analysis. This sensitivity analysis includes patients with hippocampal sclerosis but excludes those with other structural abnormalities. Group differences were assessed using one-way ANOVA with post hoc Welch's t-tests. P-values were corrected for multiple comparisons using the Benjamini-Hochberg False Discovery Rate (FDR) method.

*P < 0.05, **P < 0.001

Ipsi, ipsilateral; Contra, contralateral; CA, cornu ammonis; DG, dentate gyrus; SRLM, stratum radiatum/lacunosum-moleculare.

**Supplementary Table S23. Disease Duration Subgroup Comparison in MRI-Negative Focal Epilepsy Patients**

| **Variable** | **Shorter Duration Mean (SD)** | **Longer Duration Mean (SD)** | **P-value (ANOVA)** | **P Control vs Shorter** | **P Control vs Longer** | **P Shorter vs Longer** |
| --- | --- | --- | --- | --- | --- | --- |
| **Total Hippocampus Metrics** |  |  |  |  |  |  |
| Ipsi volume | -0.48 (1.62) | -0.85 (1.70) | <0.001** | 0.002* | <0.001** | 0.126 |
| Contra volume | -0.47 (1.50) | -0.60 (1.58) | <0.001** | 0.002* | <0.001** | 0.552 |
| Ipsi thickness | -0.01 (1.33) | -0.40 (1.34) | 0.001** | 0.939 | 0.001* | 0.051 |
| Contra thickness | 0.02 (1.22) | -0.33 (1.38) | 0.006* | 0.884 | 0.009* | 0.065 |
| Ipsi gyrification | -0.39 (1.66) | -0.62 (1.50) | <0.001** | 0.016* | <0.001** | 0.328 |
| Contra gyrification | -0.32 (1.48) | -0.37 (1.39) | 0.001** | 0.026* | 0.004* | 0.785 |
| Ipsi mean curvature | 0.20 (1.24) | 0.28 (1.75) | 0.032* | 0.097 | 0.068 | 0.747 |
| Contra mean curvature | 0.37 (1.23) | 0.19 (1.42) | 0.002* | 0.003* | 0.125 | 0.356 |
| Ipsi intrinsic curvature | 0.68 (1.84) | 0.62 (1.59) | <0.001** | <0.001** | <0.001** | 0.782 |
| Contra intrinsic curvature | 0.68 (1.78) | 0.39 (1.80) | <0.001** | <0.001** | 0.014* | 0.286 |
| **Subfield Volumes** |  |  |  |  |  |  |
| Ipsi subiculum | -0.22 (1.27) | -0.82 (1.28) | <0.001** | 0.082 | <0.001** | 0.001** |
| Contra subiculum | -0.03 (1.19) | -0.49 (1.28) | <0.001** | 0.840 | <0.001** | 0.013* |
| Ipsi CA1 | -0.48 (1.41) | -0.74 (1.57) | <0.001** | 0.001** | <0.001** | 0.261 |
| Contra CA1 | -0.54 (1.34) | -0.48 (1.53) | <0.001** | <0.001** | 0.001** | 0.782 |
| Ipsi CA2 | -0.78 (1.45) | -0.44 (2.12) | <0.001** | <0.001** | 0.014* | 0.238 |
| Contra CA2 | -0.81 (1.40) | -0.57 (1.53) | <0.001** | <0.001** | <0.001** | 0.286 |
| Ipsi CA3 | -0.29 (1.47) | -0.48 (1.80) | <0.001** | 0.043* | 0.002* | 0.411 |
| Contra CA3 | -0.28 (1.42) | -0.51 (1.67) | <0.001** | 0.043* | 0.001** | 0.328 |
| Ipsi CA4 | 0.25 (1.46) | -0.15 (1.39) | 0.015* | 0.082 | 0.236 | 0.064 |
| Contra CA4 | 0.19 (1.47) | 0.03 (1.29) | 0.220 | 0.172 | 0.772 | 0.411 |
| Ipsi DG | -0.15 (1.48) | -0.63 (1.60) | <0.001** | 0.307 | <0.001** | 0.050 |
| Contra DG | -0.10 (1.41) | -0.43 (1.54) | 0.001* | 0.502 | 0.002* | 0.126 |
| Ipsi SRLM | -0.31 (1.47) | -0.77 (1.69) | <0.001** | 0.032* | <0.001** | 0.051 |
| Contra SRLM | -0.31 (1.37) | -0.43 (1.56) | <0.001** | 0.023* | 0.002* | 0.580 |

Comparison of hippocampal Z-score metrics among healthy controls (N=424), shorter disease duration (N=152), and longer disease duration (N=160) groups in MRI-negative focal epilepsy patients. This cohort excludes all patients with hippocampal sclerosis or any other structural lesions. Patients were divided using the same disease duration median split as in the main analysis. Group differences were assessed using one-way ANOVA with post hoc Welch's t-tests. P-values were corrected for multiple comparisons using the Benjamini-Hochberg False Discovery Rate (FDR) method.

*P < 0.05, **P < 0.001

Ipsi, ipsilateral; Contra, contralateral; CA, cornu ammonis; DG, dentate gyrus; SRLM, stratum radiatum/lacunosum-moleculare.

**Supplementary Table S24. Spearman correlations between disease duration and hippocampal Z-score metrics stratified by temporal lobe epilepsy (TLE) versus non-TLE**

| **Measure** | **TLE ρ**  **(N=439)** | **TLE**  **p(FDR)** | **Non-TLE**  **ρ (N=266)** | **Non-TLE**  **p(FDR)** |
| --- | --- | --- | --- | --- |
| **Total Hippocampus Metrics** |  |  |  |  |
| Ipsi volume | -0.251 | <0.001** | -0.311 | <0.001** |
| Contra volume | 0.117 | 0.020* | -0.013 | 0.835 |
| Ipsi thickness | -0.344 | <0.001** | -0.359 | <0.001** |
| Contra thickness | -0.088 | 0.076 | -0.185 | 0.005* |
| Ipsi gyrification | -0.284 | <0.001** | -0.313 | <0.001** |
| Contra gyrification | 0.119 | 0.019* | -0.014 | 0.835 |
| Ipsi mean curvature | 0.278 | <0.001** | 0.174 | 0.009* |
| Contra mean curvature | -0.088 | 0.076 | -0.107 | 0.122 |
| Ipsi intrinsic curvature | 0.255 | <0.001** | 0.198 | 0.003* |
| Contra intrinsic curvature | -0.124 | 0.015* | -0.061 | 0.410 |
| **Subfield Volumes** |  |  |  |  |
| Ipsi subiculum | -0.338 | <0.001** | -0.354 | <0.001** |
| Contra subiculum | -0.070 | 0.154 | -0.168 | 0.011* |
| Ipsi CA1 | -0.312 | <0.001** | -0.300 | <0.001** |
| Contra CA1 | 0.143 | 0.005* | 0.042 | 0.544 |
| Ipsi CA2 | -0.171 | <0.001** | -0.129 | 0.060 |
| Contra CA2 | 0.170 | <0.001** | 0.117 | 0.089 |
| Ipsi CA3 | -0.290 | <0.001** | -0.300 | <0.001** |
| Contra CA3 | 0.108 | 0.032* | -0.077 | 0.284 |
| Ipsi CA4 | -0.335 | <0.001** | -0.212 | 0.001* |
| Contra CA4 | 0.008 | 0.868 | -0.051 | 0.461 |
| Ipsi DG | -0.353 | <0.001** | -0.357 | <0.001** |
| Contra DG | 0.053 | 0.282 | -0.102 | 0.137 |
| Ipsi SRLM | -0.340 | <0.001** | -0.358 | <0.001** |
| Contra SRLM | 0.099 | 0.047* | -0.058 | 0.414 |

Spearman rank correlation coefficients (ρ) between disease duration and hippocampal global and subfield Z-score metrics, stratified by temporal lobe epilepsy (TLE) and non-TLE. P values were adjusted for multiple comparisons using the Benjamini–Hochberg false discovery rate (FDR) procedure, applied independently within each subgroup.
Ipsi, ipsilateral; Contra, contralateral; CA, cornu ammonis; DG, dentate gyrus; SRLM, stratum radiatum/lacunosum-moleculare.

**Supplementary Table S25. Post-hoc power and minimum detectable effect (MDE) for the primary cross-sectional endpoint in key prespecified subgroups**

| **Subgroup** | **N** | **Spearman ρ** | **P** | **P_FDR_** | **Achieved Power (%)** | **MDE (\|ρ\| for 80% power)** |
| --- | --- | --- | --- | --- | --- | --- |
| Non-HS patients | 494 | -0.13 | 0.004 | 0.042 | 82.4 | 0.126 |
| MRI-negative patients | 312 | -0.138 | 0.015 | 0.051 | 68.4 | 0.158 |
| Pediatric patients (<18 years) | 257 | -0.053 | 0.4 | 0.838 | 13.4 | 0.174 |

This table summarizes subgroup-specific post-hoc power analyses for the primary cross-sectional endpoint, defined as the association between disease duration and ipsilateral hippocampal volume. Achieved power was calculated at the observed Spearman correlation coefficient (ρ), and the minimum detectable effect (MDE) was defined as the smallest absolute correlation yielding 80% power at two-sided α = 0.05.

**Figure legends**

**Supplementary Figure S1. Participant Flow Diagram for Study Enrollment and Inclusion**

Flow diagram showing recruitment, screening, and inclusion/exclusion of participants in the study.
A total of 848 patients with focal epilepsy and 430 healthy controls were initially recruited across three centers. After exclusion for unknown or bilateral seizure focus and unsatisfactory HippUnfold segmentation, 705 patients with focal epilepsy and 424 healthy controls were included in the final analysis.

**Supplementary Figure S2. Normality assessment of residuals from statistical models for hippocampal morphological features.**

Figure legends: Each panel (A–M) shows a histogram (left) and Q-Q (Quantile-Quantile) plot (right) of the residuals for one hippocampal morphological metric in the healthy control cohort. Panels A–E correspond to global hippocampal metrics; panels F–M show subfield volumes, as detailed in Methods (Section 2.3 Segmentation and Feature Extraction). The bell-shaped distributions and the close fit of the Q-Q plots to the diagonal line (red) indicate that model residuals are approximately normally distributed. The cyst volume (panel M) deviated from normality and was excluded from main group comparisons.

Hipp, hippocampal; MeanCurv, mean curvature; IntrinsicCurv, intrinsic curvature; Thickness, cortical thickness; CA, cornu ammonis subfield; DG, dentate gyrus; SRLM, stratum radiatum/lacunosum-moleculare; Q-Q, quantile-quantile.

**Supplementary Figure S3. Complete group comparisons of hippocampal subfield Z-scores by disease duration**
Boxplots with overlaid individual data points show the distribution of hippocampal Z-scores for global and subfield metrics across three groups: healthy controls, patients with shorter disease duration (duration < median), and patients with longer disease duration (duration ≥ median). Panels (A–X) represent ipsilateral (Ipsi) and contralateral (Contra) hippocampal features, as labeled. Group comparisons were performed using one-way ANOVA with post hoc Welch’s t-tests; p-values were corrected for multiple comparisons using the Benjamini-Hochberg False Discovery Rate (FDR) method. p-values shown are FDR-adjusted.

*: p < 0.05, **: p < 0.001.
Ipsi, ipsilateral; Contra, contralateral; CA, cornu ammonis subfield; DG, dentate gyrus; SRLM, stratum radiatum/lacunosum-moleculare.

**Supplementary Figure S4. Complete longitudinal trajectories of hippocampal features**

Percentile curves (5th, 25th, 50th, 75th, 95th) derived from healthy controls illustrate the expected age-related distribution for each hippocampal feature. Colored points mark each patient’s Scan 1 and Scan 2, connected by lines to indicate within-subject change. This figure provides full results for all hippocampal global and subfield features, complementing the representative set shown in Figure 4.

All volume metrics are in mm³.

Hipp, hippocampal; CA, cornu ammonis subfield; DG, dentate gyrus; SRLM, stratum radiatum/lacunosum-moleculare; LOWESS, locally weighted scatterplot smoothing; IQR, interquartile range.

**Supplementary Figure S5. Distribution of inter-scan intervals and individual longitudinal slopes for hippocampal Z-scores**

(A) Histogram showing the distribution of inter-scan intervals for the 80 patients in the longitudinal sub-cohort (mean = 1.36 ± 1.06 years; median = 0.90 years). (B–D) Histograms showing the distribution of patient-specific annual slopes (Z-score change per year) extracted from the random effects of linear mixed-effects models for ipsilateral hippocampal volume (B), contralateral hippocampal volume (C), and contralateral cortical thickness (D). Red dashed lines indicate the group mean slope; orange dashed lines indicate the median.
