# Supplementary material for: Duration-dependent hippocampal structural changes in focal epilepsy: multicenter neuroimaging evidence
Source: J Transl Med. 2026 May 8;24:843. doi: 10.1186/s12967-026-08230-x (PMC13326556; doi:10.1186/s12967-026-08230-x)
Supplement: Supplementary file 2 — Supplementary Material 2 [file 12967_2026_8230_MOESM2_ESM.pdf]

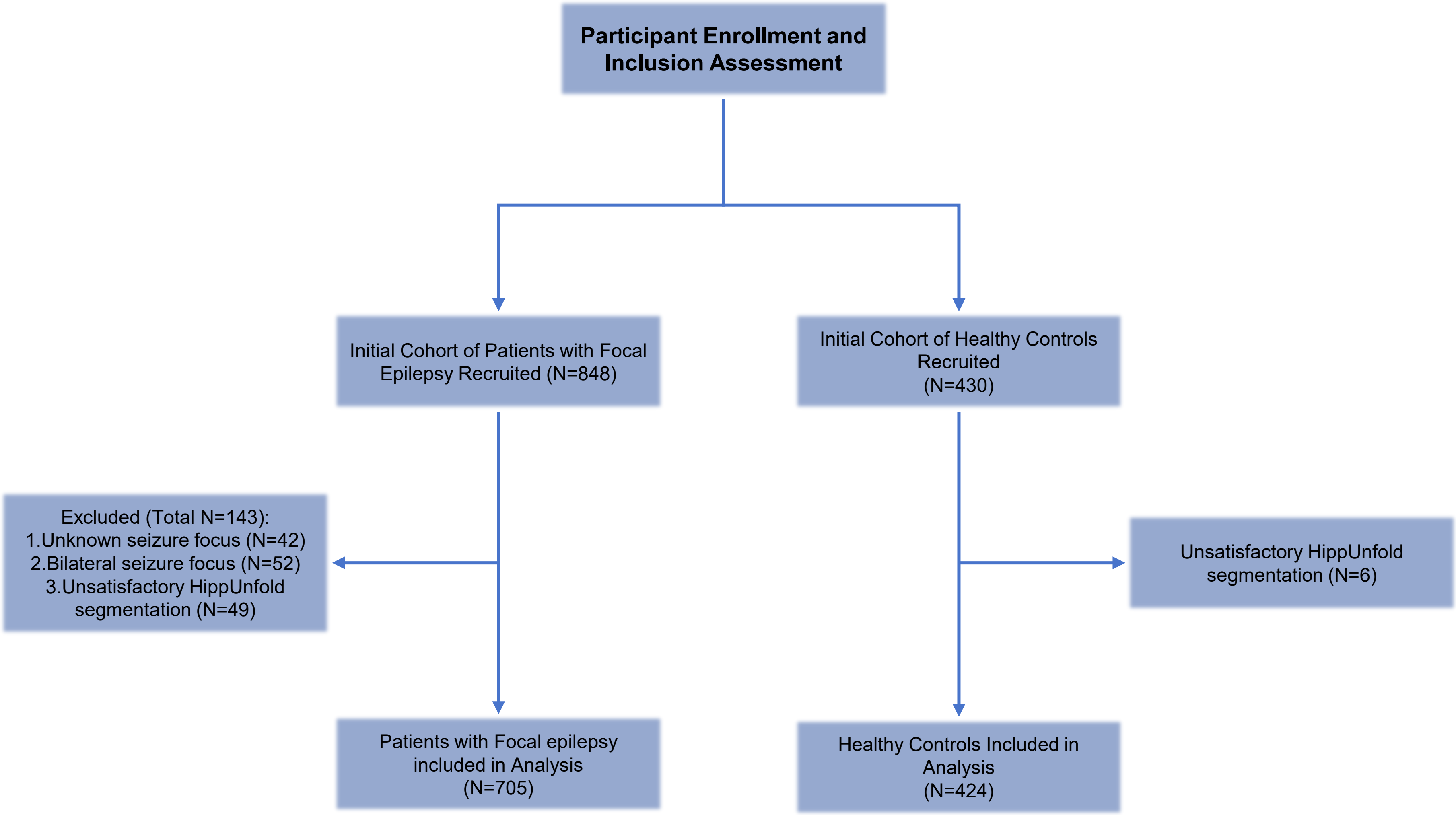
Participant Enrollment and Inclusion Assessment
Initial Cohort of Patients with Focal Epilepsy Recruited (N=848)
Initial Cohort of Healthy Controls Recruited (N=430)
Excluded (Total N=143):
1.Unknown seizure focus (N=42)
2.Bilateral seizure focus (N=52)
3.Unsatisfactory HippUnfold segmentation (N=49)
Unsatisfactory HippUnfold segmentation (N=6)
Patients with Focal epilepsy included in Analysis (N=705)
Healthy Controls Included in Analysis (N=424)
