# Supplementary figures and images for: Duration-dependent hippocampal structural changes in focal epilepsy: multicenter neuroimaging evidence
Source: J Transl Med. 2026 May 8;24:843. doi: 10.1186/s12967-026-08230-x (PMC13326556; doi:10.1186/s12967-026-08230-x)

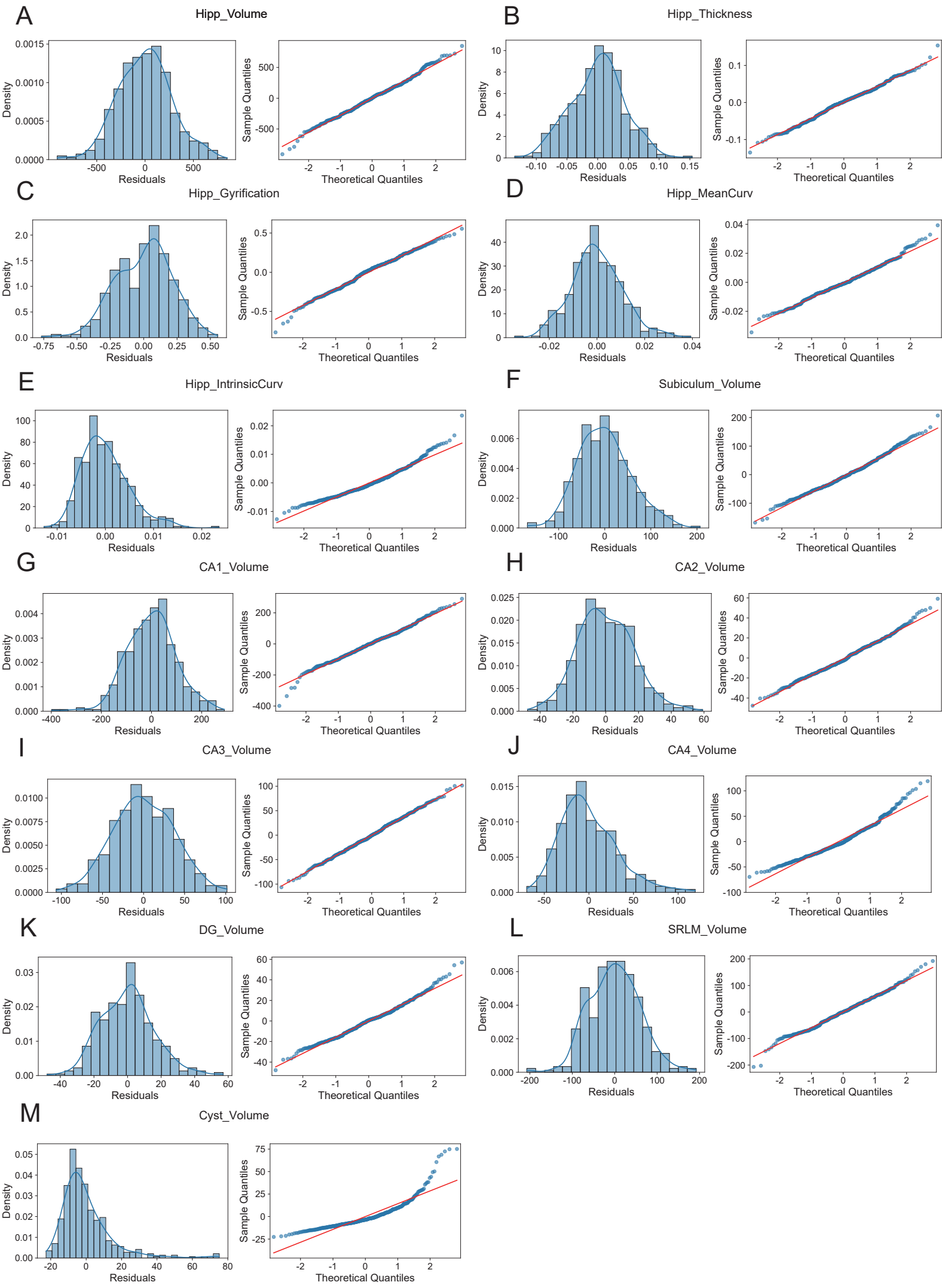

Supplement: Supplementary file 3 — Supplementary Material 3 [file 12967_2026_8230_MOESM3_ESM.pdf]

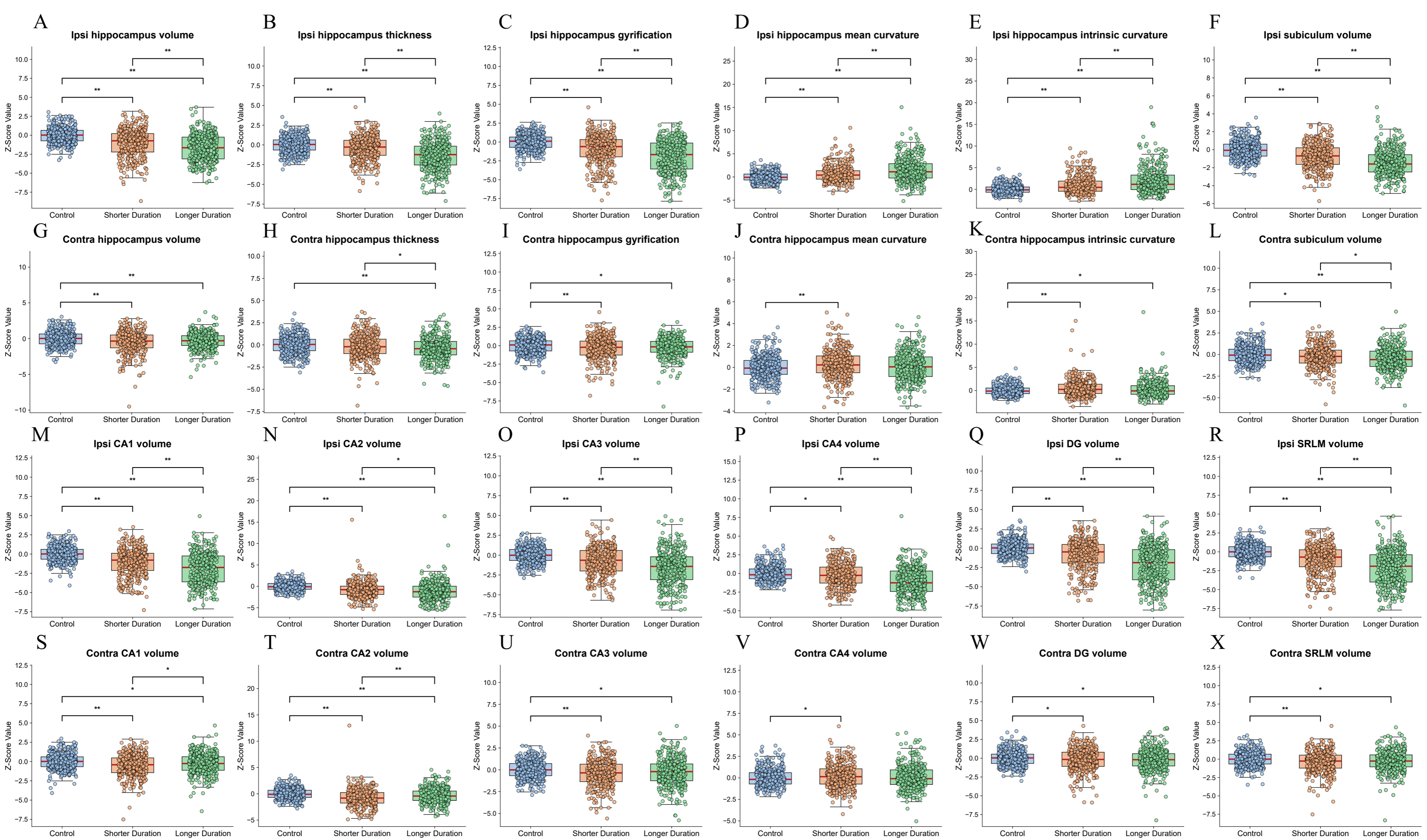

Supplement: Supplementary file 4 — Supplementary Material 4 [file 12967_2026_8230_MOESM4_ESM.pdf]

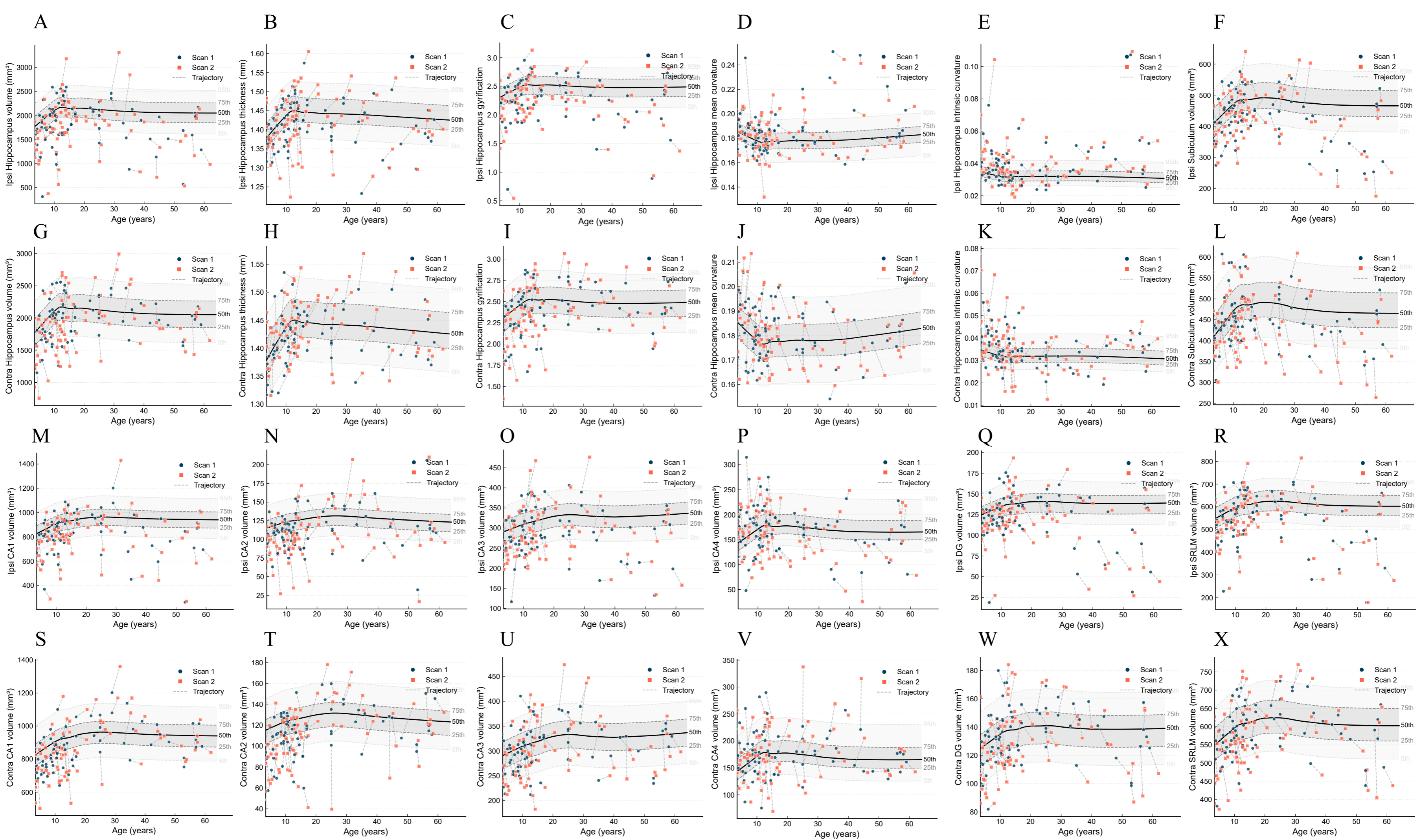

Supplement: Supplementary file 5 — Supplementary Material 5 [file 12967_2026_8230_MOESM5_ESM.pdf]

**A**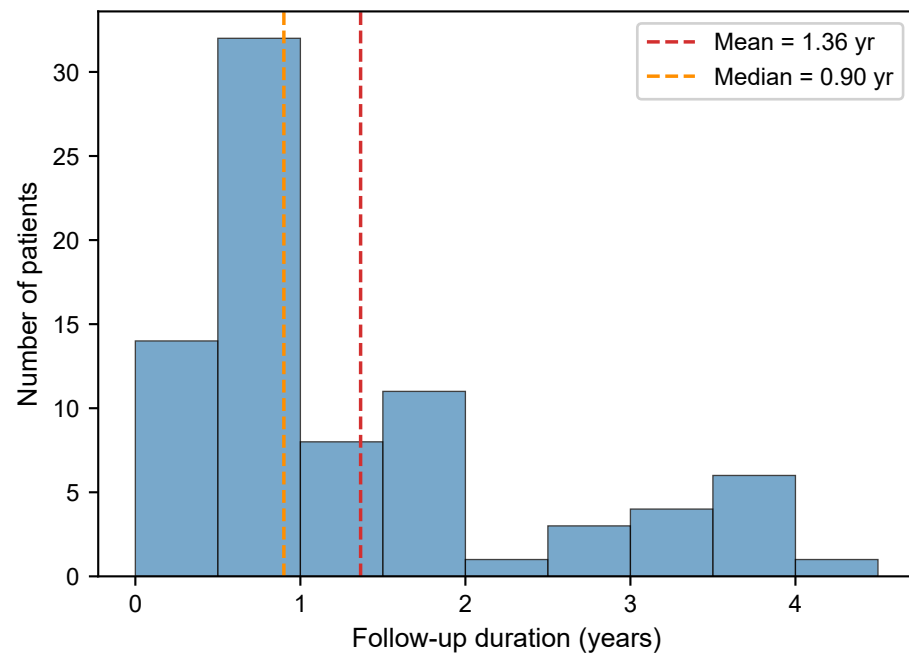**B**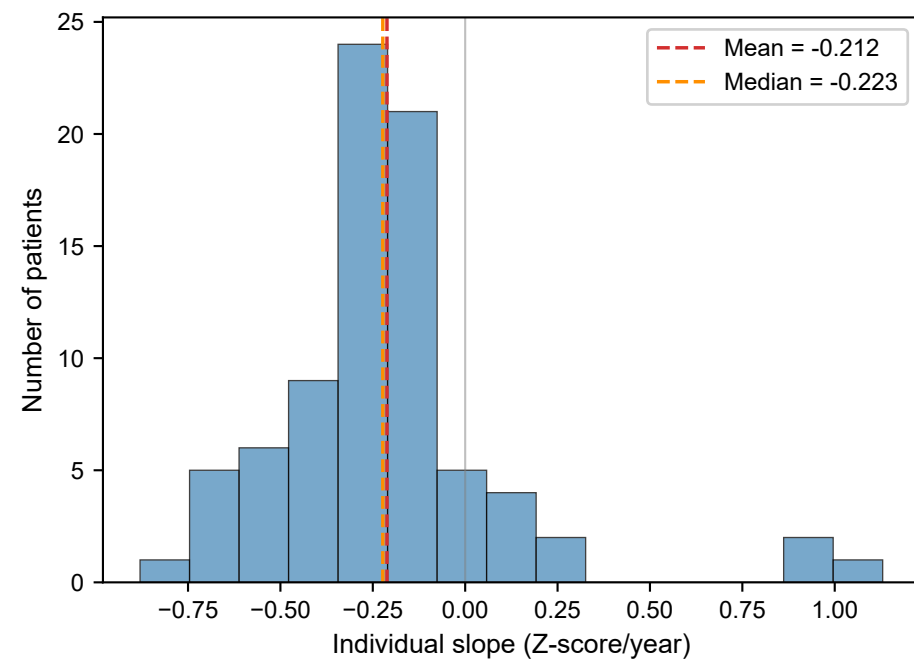**C**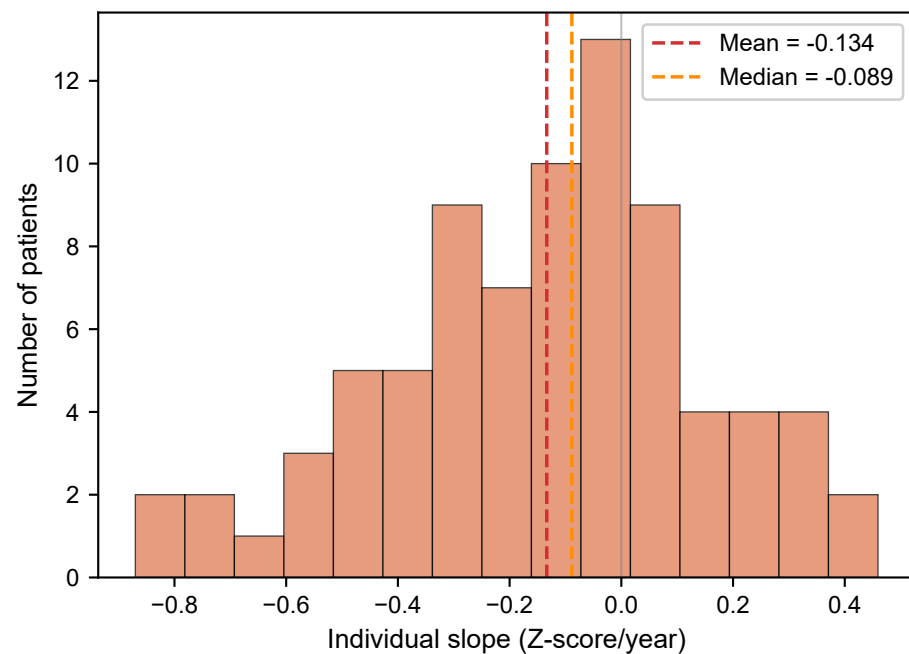**D**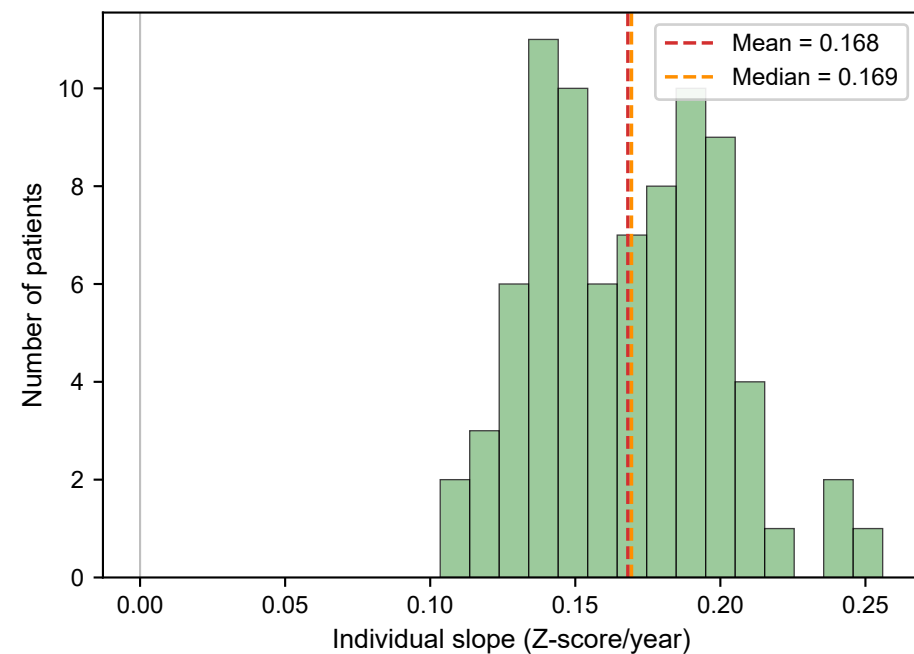

Supplement: Supplementary file 6 — Supplementary Material 6 [file 12967_2026_8230_MOESM6_ESM.pdf]
